# Tumor Electric Field Therapy Inhibits Epithelial‐Mesenchymal Transition, Invasion, and Migration of Glioblastoma by Targeting the c‐FOS/CXCL14 Axis

**DOI:** 10.1002/cns.70926

**Published:** 2026-05-19

**Authors:** Cheng Sun, Yuyang Liu, Junyi Chen, Jie Pei, Chao Li, Jinxin Lan, Er Wen, Jialin Liu, Ze Li, Ling Chen

**Affiliations:** ^1^ Medical School of Chinese PLA Beijing China; ^2^ Department of Neurosurgery Chinese PLA General Hospital Beijing China; ^3^ Ninth Medical Center of Chinese PLA General Hospital Beijing China; ^4^ Department of Neurosurgery 920th Hospital of Joint Logistics Support Force Kunming China; ^5^ Graduate School Kunming Medical University Kunming China; ^6^ Department of Neurosurgery, the Second Affiliated Hospital Chongqing Medical University Chongqing China; ^7^ Department of Neurosurgery, Xuanwu Hospital Capital Medical University Beijing China; ^8^ School of Medicine Nankai University Tianjin China

**Keywords:** c‐FOS, CXCL14, epithelial‐mesenchymal transition, glioblastoma, tumor electric field therapy

## Abstract

**Background:**

Glioblastoma (GBM) is among the most aggressive and treatment‐resistant primary brain tumors. The mesenchymal subtype of GBM shows a particularly unfavorable prognosis. Epithelial‐mesenchymal transition (EMT) is a critical phenotypic characteristic of this subtype. Tumor electric field therapy (TEFT) has emerged as a promising adjuvant therapy, but its underlying anti‐GBM mechanisms remain incompletely elucidated.

**Methods:**

Key molecular targets of TEFT were identified through integrated multi‐omics data analysis. U87, U251, and T98G cell lines received TEFT treatment at 200 kHz and 2.2 V/cm for 72 h. Stable cell models with CXCL14 and c‐FOS knockdown or overexpression were established using lentiviral vectors. Cellular phenotypes were assessed via wound healing assays, transwell migration and invasion assays, and western blot analysis. The regulatory hierarchy between c‐FOS and CXCL14 was verified via chromatin immunoprecipitation (ChIP) assays and rescue experiments. Mechanistic insights were validated in orthotopic nude mouse models and clinical patient specimens.

**Results:**

CXCL14 was identified by integrated bioinformatics analysis. It showed significant overexpression in mesenchymal subtypes and was strongly associated with poor prognosis. Single‐cell sequencing analysis suggested a significant increase in the EMT score of the subgroup with high CXCL14 expression. TEFT induced a morphological shift from mesenchymal to epithelial‐like characteristics. It downregulated mesenchymal markers, including N‐cadherin, Vimentin, and Snail, while upregulating E‐cadherin. Mechanistic investigations demonstrated that TEFT promoted the degradation of c‐FOS, leading to CXCL14 downregulation and subsequent inhibition of EMT. In vivo experiments confirmed the critical role of the c‐FOS/CXCL14 axis in regulating GBM invasion and migration potential.

**Conclusion:**

This study first revealed a novel mechanism which TEFT suppressed EMT in GBM via the c‐FOS/CXCL14 axis. These findings provided a new therapeutic target and a theoretical foundation for optimizing TEFT efficacy.

## Graphical abstract

Integrated multi‐omics screening identified CXCL14 as a TEFT target. Functional validation via knockdown/overexpression in GBM cells confirmed its role in migration, invasion, and EMT. Mechanism studies revealed TEFT suppresses EMT via the c‐FOS/CXCL14 axis, validated in orthotopic mouse models and clinical samples.

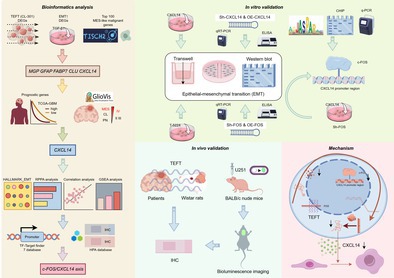

**Abbreviations**
AP‐1: activator protein‐1
CHIP: chromatin immunoprecipitation
CL: classical
CNS: central nervous system
DEGs: differentially expressed genes
DMEM: Dulbecco's Modified Eagle Medium
ECM: extracellular matrix
ELISA: enzyme‐linked immunosorbent assay
EMT: epithelial‐mesenchymal transition
FBS: fetal bovine serum
GBM: glioblastoma
GEO: gene expression omnibus
HE: hematoxylin and eosin
IHC: immunohistochemistry
MES: mesenchymal
NC: negative control
NCBI: National Center for Biotechnology Information
NCCN: National Comprehensive Cancer Network
OS: overall survival
PN: proneural
qRT‐PCR: quantitative real‐time PCR
SD: standard deviation
ShRNA: short hairpin RNA
TCGA: the Cancer Genome Atlas
TEFT: tumor electric field therapy
TF: transcription factor
TGF‐β: transforming growth factor‐beta
TMZ: temozolomide
WB: western blot

## 1 Introduction

Glioblastoma (GBM) is the most common primary malignant tumor of the central nervous system, accounting for 14.2% of all CNS tumors and 50.9% of malignant CNS tumors. The annual mortality rate is approximately 4.42 per 100,000, with 17,206 deaths annually. The 5‐year relative survival rate for CNS malignancies is 35.7%, while GBM patients have a median survival of only 8 months and a 5‐year survival rate as low as 6.9% [1]. The standard Stupp regimen, which combines surgical resection, postoperative concurrent chemoradiotherapy, and 6‐month temozolomide (TMZ) treatment, has significantly improved patient survival [2]. However, GBM prognosis remains poor, prompting ongoing research into new treatments. Clinical studies have demonstrated that the combination of bevacizumab with TMZ and postoperative chemoradiotherapy failed to significantly improve overall survival (OS), while inducing adverse effects including hypertension, venous thrombosis, delayed wound healing, and infections [3]. Similarly, carmustine wafers have not been incorporated into standard GBM treatment protocols due to their limited efficacy and associated risks of intracranial hypertension, infections, and impaired wound healing [4, 5]. Notably, molecular targeted therapies have predominantly failed to achieve expected outcomes in clinical trials over the past decade [6]. These clinical challenges underscore the urgent need to develop novel non‐invasive and effective therapeutic strategies.

Tumor electric field therapy (TEFT), as a novel therapeutic approach following surgery, radiotherapy, and chemotherapy, uses low‐intensity, intermediate‐frequency alternating electric fields to inhibit tumor cell proliferation. Our previous research showed TEFT can effectively slow GBM growth in vitro and in vivo [7]. Clinical trials demonstrate that TEFT combined with TMZ achieves a median OS of 20.9 months, significantly better than chemotherapy alone. Both the National Comprehensive Cancer Network (NCCN) and the Chinese Glioma Committee have included TEFT in treatment guidelines as a standard therapy for GBM [8, 9]. Studies indicate TEFT not only provides superior survival benefits but also has better safety and less impact on quality of life compared to conventional chemotherapy. With increasing clinical application of TEFT, research has gradually focused on elucidating its anti‐tumor mechanisms and optimizing clinical combination strategies. A previous study of our team demonstrated TEFT could remodel the extracellular matrix (ECM) of GBM by downregulating COL6A1 expression and inhibiting the focal adhesion pathway [10]. However, the underlying mechanisms of TEFT related anti‐GBM require further investigation.

Epithelial‐mesenchymal transition (EMT) is a process where cells lose polarity and intercellular adhesion, altering expression of cell surface and cytoskeletal proteins to acquire migratory properties [11]. In cancer, EMT represents a dynamic process where epithelial cells transform into mesenchymal‐like cells, gaining stem cell‐like characteristics, increased motility and invasiveness, resistance to therapies, as well as immune evasion and immunosuppressive properties [12]. Recently, Wang et al. molecularly classified GBM patients into three groups: proneural (PN), mesenchymal (MES), and classical (CL). The MES subtype represents the most aggressive form of GBM, characterized by pronounced tumor heterogeneity, immunosuppressive microenvironment [13], therapeutic resistance [14] and the poorest patient prognosis [15]. However, research on this subtype features and response to TEFT remains relatively inadequate, which may contribute to the low success rate in translating basic GBM research into clinical applications [14]. Moreover, EMT‐related genes demonstrate significant correlations with MES‐like GBM characteristics [16]. Although GBM does not originate from epithelial cells, making the term “EMT” technically imprecise in this context, studies have revealed that modulation of classical EMT markers can induce GBM cells to transition into the MES subtype [17, 18, 19, 20]. Currently, there is a lack of research on the impact of TEFT on EMT processes in GBM.

CXCL14 is one of the highly conserved chemokines constitutively expressed in skin epithelium [21]. As a member of the CXC chemokine subfamily, CXCL14 mediates immune cell infiltration, dendritic cell maturation, upregulation of major histocompatibility complex (MHC)‐I expression, and cell mobilization [22]. Numerous studies have demonstrated that CXCL14 plays a significant role in the EMT process across various cancers. Research has shown its association with lymph node metastasis in papillary thyroid carcinoma [23], its ability to promote migration and invasion of colorectal cancer cells [24], and its overexpression and secretion by cancer‐associated fibroblasts (CAFs) that facilitates tumor growth and invasion in both breast and prostate cancers [25, 26]. These findings collectively establish CXCL14 as an important regulator in tumor EMT processes and malignant progression. Activator protein‐1 (AP‐1), a transcription factor (TF) discovered in the 1990s, primarily consists of JUN and FOS subfamilies which share a conserved domain mediating DNA binding. As a FOS family member, c‐FOS plays a crucial role in tumor progression; existing studies have demonstrated that c‐FOS plays a crucial role in maintaining cancer stem cells (CSCs) in GBM and head and neck squamous cell carcinomas [27, 28]. Furthermore, research has revealed its involvement in activating EMT and promoting metastasis in breast cancer [29].

Our research suggested that CXCL14 was regulated in MES subtypes and correlated with the EMT process and worse prognosis. Prediction of TF combined with chromatin immunoprecipitation (CHIP) revealed that c‐FOS might be the potential upstream of CXCL14. Furthermore, TEFT may exert important effects on GBM EMT by influencing the c‐FOS/CXCL14 axis, thereby affecting its invasive and migratory phenotypes to achieve therapeutic outcomes in vivo and in vitro. Regulating the c‐FOS/CXCL14 signaling axis or its downstream effector molecules may be the core mechanism of TEFT related to anti‐GBM.

## 2 Methods

A comprehensive description of all experimental procedures, materials, and analytical methods is provided in the Supporting Information Methods document [30, 31, 32, 33, 34, 35, 36, 37].

## 3 Result

### 3.1 TEFT Suppressed GBM Migration, Invasion, and EMT

**FIGURE 1:**
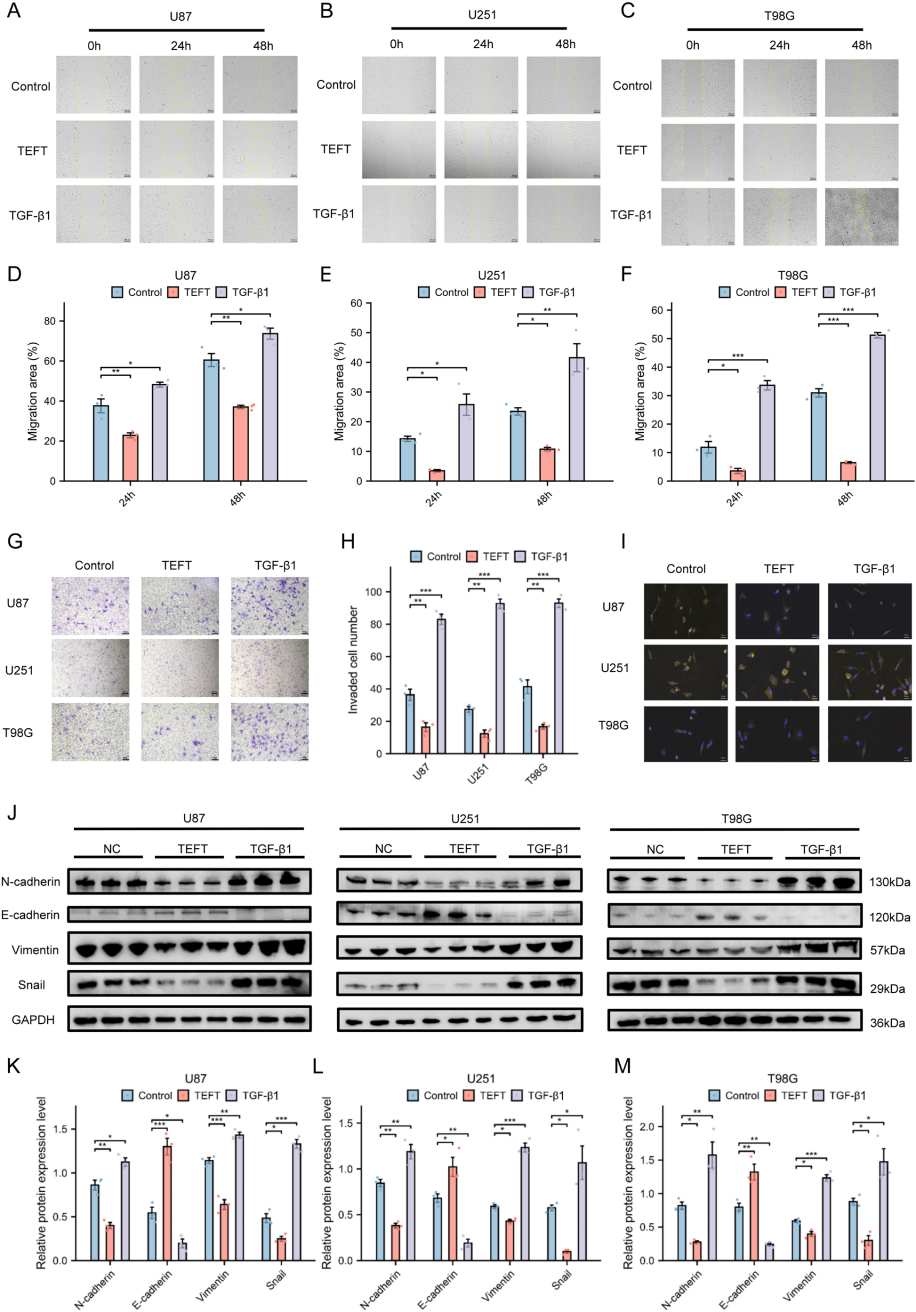
TEFT suppressed GBM migration, invasion, and EMT, whereas TGF‐β1 promotes these malignant processes. (A–F) Wound healing assay and statistical analysis of cell lines following TEFT and TGF‐β1 treatment, One‐way ANOVA with Tukey's post hoc test, *n* = 3, *p < 0.05, **p < 0.01, ***p < 0.001. Scale bar = 200 μm. (G, H) Cell invasion assay and statistical analysis following TEFT and TGF‐β1 treatment, one‐way ANOVA with Tukey's post hoc test, *n* = 3, *p < 0.05, **p < 0.01, ***p < 0.001. Scale bar = 100 μm. (I) Cellular morphology following TEFT and TGF‐β1 treatment (Fluorescence Staining): Epithelial‐like transformation induced by TEFT versus mesenchymal‐like transition promoted by TGF‐β1. Scale bar = 50 μm. (J–M) WB analysis of mesenchymal markers (N‐cadherin, Vimentin, and Snail) and epithelial marker (E‐cadherin) expression following TEFT and TGF‐β1 treatment, one‐way ANOVA with Tukey's post hoc test, *n* = 3, *p < 0.05, **p < 0.01, ***p < 0.001.

Our experimental data demonstrated that TEFT significantly inhibited the migratory capacity of U87, U251, and T98G GBM cell lines in a wound healing assay (U87: 24 h *p* < 0.01, 48 h *p* < 0.01; U251: 24 h *p* < 0.05, 48 h *p* < 0.05; T98G: 24 h *p* < 0.05, 48 h *p* < 0.001) (Figure 1A–F). Furthermore, transwell migration and invasion assays confirmed that TEFT‐exposed cells exhibited markedly reduced migration and invasion abilities compared to the control group (U87: *p* < 0.01; U251: *p* < 0.01; T98G: *p* < 0.01) (Figure 1G,H). IF staining revealed TEFT induced significant morphological changes in GBM cells, demonstrating characteristic mesenchymal‐to‐epithelial transition‐like (MET‐like) features [38]. Specifically, the treated cells transitioned from an originally elongated, pseudopod‐rich mesenchymal morphology (typical EMT state) to a more rounded, epithelial‐like morphology with reduced pseudopodia formation [19, 39] (Figure 1I). WB analysis indicated that TEFT exposure significantly downregulated the expression of mesenchymal markers (N‐cadherin Vimentin, and Snail) while upregulating the epithelial marker E‐cadherin in all three cell lines (All comparisons *p* < 0.05; see Table S3 for complete statistical details) (Figure 1J–M) (Antibodies used can be found in Table S2). EMT is a biological process through which polarized epithelial cells acquire mesenchymal characteristics, thereby enhancing tumor cell migration and invasion. To further validate our findings, we established an EMT phenotype enhanced by stimulating cells with TGF‐β1 for 48 h (10 ng/mL) [40, 41]. Subsequent experiments yielded contrasting results to those observed in the TEFT group, confirming that TGF‐β1 promotes GBM cell migration, invasion, and EMT while upregulating the epithelial marker E‐cadherin in all three cell lines (All comparisons *p* < 0.05; see Table S3 for complete statistical details) (Figure 1A–M). Collectively, these findings suggested that TEFT might effectively suppress GBM cell migration, invasion, and EMT, whereas TGF‐β1 stimulation exerts the opposite effect.

### 3.2 CXCL14 as a Potential Key Regulator of EMT in Glioblastoma

**FIGURE 2:**
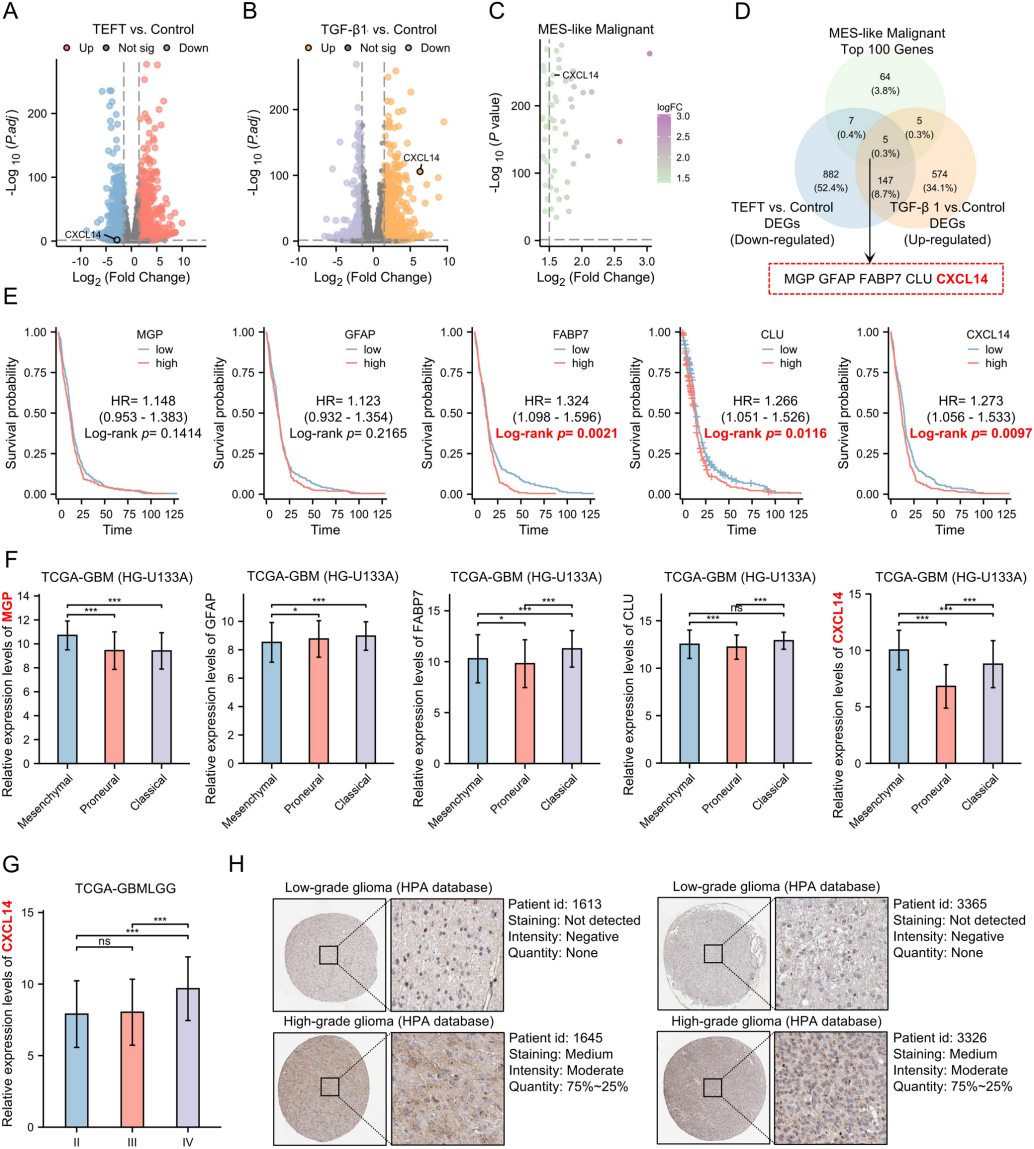
CXCL14 as a potential key regulator of EMT in GBM. (A) Volcano plot presented 1041 down‐regulated and 954 up‐regulated DEGs in U251 cells exposed on TEFT. (B) Volcano plot presented 292 down‐regulated and 731 up‐regulated DEGs in U251 cells exposed on TGF‐β1. (C) Volcano plot illustrated the top 100 MES‐like malignant genes ranked by fold change. (D) Five candidate genes screened by venn plot. (E) Survival analyses of five candidate genes in TCGA‐GBM (HU133A), Log‐rank test. (F) Expression patterns of five genes across mesenchymal, proneural and classical GBM. Data are mean ± standard deviation (SD), Kruskal‐Wallis test, and Dunn's post hoc test. ^ns^ *p* ≥ 0.05, *p < 0.05, ***p < 0.001. (G) Expression profile of CXCL14 in different grades of glioma (TCGA‐GBM HU133A). Data are mean ± SD, Kruskal‐Wallis test, and Dunn's post hoc test. ^ns^ *p* ≥ 0.05, ***p < 0.001. (H) IHC staining of CXCL14 in low‐grade and high‐grade glioma derived from HPA database.

DEGs analysis among the control group, TEFT‐treated, and TGF‐β1‐treated groups revealed numerous DEGs (TEFT vs. Control: 1041 Down‐regulated DEGs; 954 Up‐regulated DEGs; TGF‐β1 vs. Control: 292 Down‐regulated DEGs; 731 Up‐regulated DEGs) (Figure 2A,B). The top 100 mesenchymal‐like (MES‐like) malignant genes downloaded from the TISCH2 database were presented in a volcano plot (Figure 2C). By intersecting down‐regulated DEGs in the TEFT‐treated group with up‐regulated DEGs in the TGF‐β1‐treated group and MES‐like malignant genes, we identified five candidate genes (MGP, GFAP, FABP7, CLU, and CXCL14) (Figure 2D). Survival analysis revealed FABP7 [HR = 1.324 (1.098–1.596) Log‐rank *p* = 0.0021], CLU [HR = 1.266 (1.051–1.526) Log‐rank *p* = 0.0116], and CXCL14 [HR = 1.273 (1.056–1.533) Log‐rank *p* = 0.0097] were associated with bad prognosis of GBM (Figures 2E and S1D). Expression analysis demonstrated the levels of MGP and CXCL14 were highest in the MES subtypes compared to the PN and CL subtypes (Figures 2F and S1C). Additionally, Ivy GAP data showed CXCL14 upregulation in microvascular proliferative and cellular tumor regions versus the lesion edge (Figure S1B). Moreover, relative expression levels of CXCL14 were highest in grade IV glioma compared with grade II and III glioma (Figure 2G). IHC staining further confirmed elevated CXCL14 protein levels in high‐grade glioma relative to low‐grade glioma (Figures 2H and S1A).

ScRNA sequencing data indicated that CXCL14‐high subpopulations were mainly enriched in EMT‐high clusters (Figures S2A–E and S3A,B). Additionally, the RPPA analysis revealed elevated expression of EMT‐related proteins (Paxillin, Fibronectin, and Collagen VI) in CXCL14‐high groups (Figure S3C). Based on proteogenomic data of GBM, association analyses showed that CXCL14 protein levels were positively correlated with PROGENy TGF‐β1 (Rho = 0.34, *p* = 6.70 × 10^−4^), HALLMARK_TGF_BETA_SIGNALING (Rho = 0.24, *p* = 0.016), tumor size (Rho = 0.35, *p* = 3.50 × 10^−4^), stromal score calculated by the ESTIMATE algorithm (Rho = 0.40, *p* = 4.80 × 10^−5^), stromal score calculated by the Xcell algorithm (Rho = 0.32, *p* = 1.20 × 10^−3^), HALLMARK_APICAL_JUNCTION (Rho = 0.37, *p* = 1.60 × 10^−4^), HALLMARK_COAGULATION (Rho = 0.42, *p* = 2.20 × 10^−5^), and HALLMARK_EMT (Rho = 0.50, *p* = 2.70 × 10^−7^). In contrast, CXCL14 protein levels exhibited negative correlations with tumor purity determined by the ABSOLUTE method (Rho = −0.32, *p* = 1.50 × 10^−3^) and tumor purity determined by whole‐genome sequencing (WGS) (Rho = −0.28, *p* = 4.80 × 10^−3^) (Figure S3D). Furthermore, GSEA indicated that CXCL14 protein expression was significantly associated with EMT in both GBM (HALLMARK_EMT: set size = 173, leading edge number = 92, enrichment score (ES) = 0.7797, normalized enrichment score (NES) = 3.1574, *p* < 2.2 × 10^−16^), false discovery rate (FDR) < 2.2 × 10^−16^ and pan‐cancer contexts (HALLMARK_EMT: set size = 193, leading edge number = 114, ES = 0.82825, NES = 2.9136, *p* < 2.2 × 10^−16^, FDR < 2.2 × 10^−16^) (Figure S3E,F). Taking together, these results strongly suggest that CXCL14 may serve as a critical regulator of EMT in GBM.

### 3.3 CXCL14 Promoted Migration, Invasion, and EMT in GBM

**FIGURE 3:**
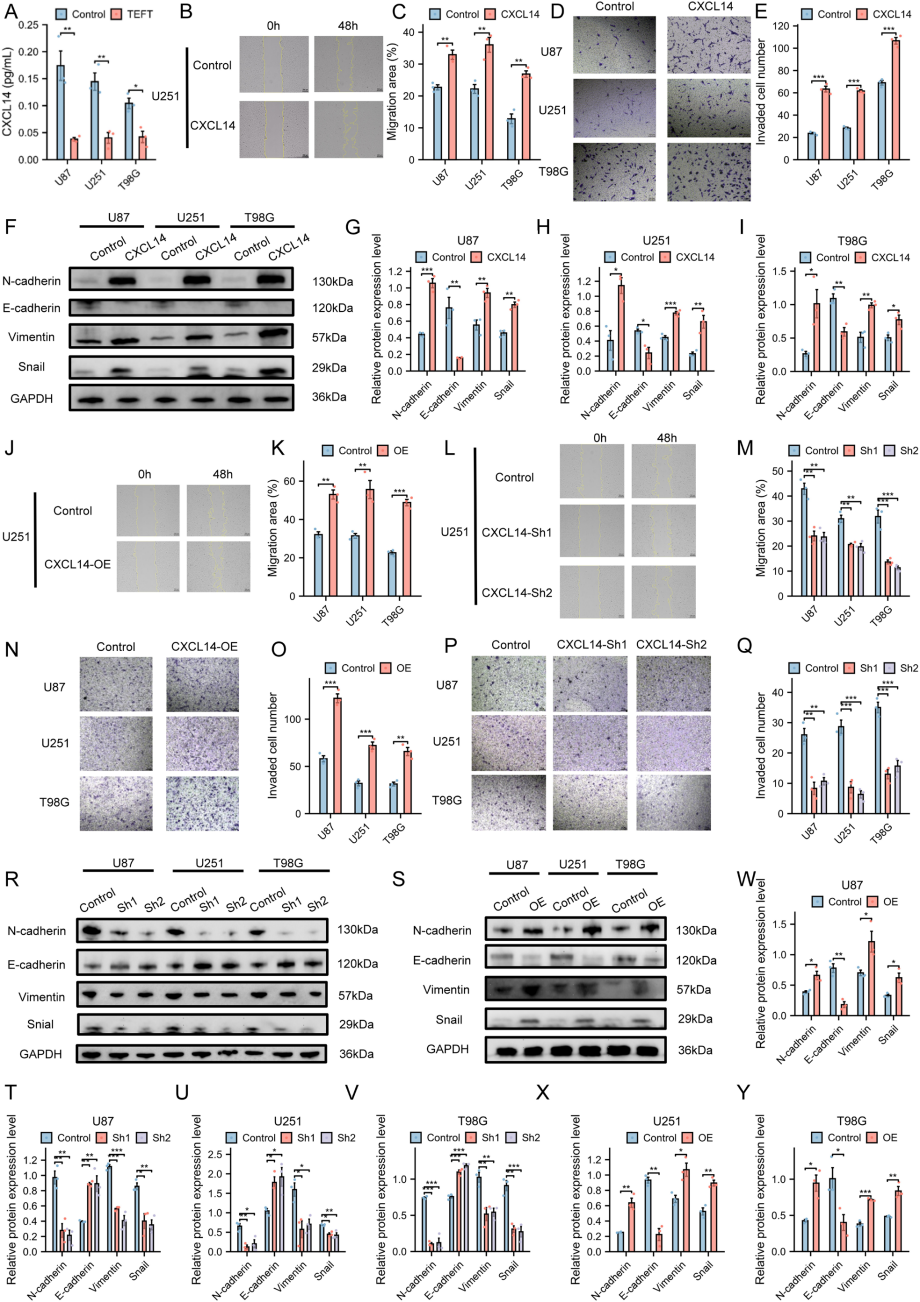
CXCL14 promoted migration, invasion, and EMT in GBM. (A) Concentration of CXCL14 in cell culture supernatant following TEFT, Student's *t*‐test, *n* = 3, *p < 0.05, **p < 0.01, ***p < 0.001. (B, C) Wound healing assay of U251 cell line following exogenous CXCL14 treatment and statistical analysis across three cell lines, Student's *t*‐test, *n* = 3, *p < 0.05, **p < 0.01, ***p < 0.001. Scale bar = 200 μm. (D, E) Cell invasion assay and statistical results of three cell lines following exogenous CXCL14 treatment, Student's *t*‐test, *n* = 3, *p < 0.05, **p < 0.01, ***p < 0.001. Scale bar = 100 μm. (F–I) WB analysis and statistical results of three cell lines following exogenous CXCL14 treatment, Student's *t*‐test, *n* = 3, *p < 0.05, **p < 0.01, ***p < 0.001. (J–M) Wound healing assay in U251 cells following CXCL14 overexpression and knockdown with statistical analysis across three cell lines, Student's *t*‐test for two groups, one‐way ANOVA with Tukey's post hoc test for three groups, *n* = 3, *p < 0.05, **p < 0.01, ***p < 0.001. Scale bar = 200 μm. (N–Q) Invasion assay and statistical analysis of three cell lines following CXCL14 overexpression and knockdown, Student's *t*‐test for two groups, one‐way ANOVA with Tukey's post hoc test for three groups, *n* = 3, *p < 0.05, **p < 0.01, ***p < 0.001. Scale bar = 100 μm. (R–Y) WB analysis and statistical analysis of three cell lines following CXCL14 overexpression and knockdown, Student's *t*‐test for two groups, one‐way ANOVA with Tukey's post hoc test for three groups, *n* = 3, *p < 0.05, **p < 0.01, ***p < 0.001.

First, we measured the levels of CXCL14 in the supernatant of TEFT‐exposed cells using ELISA, and found that they were significantly lower compared to untreated cells. (U87: *p* < 0.01; U251: *p* < 0.01; T98G: *p* < 0.05) (Figure 3A), Then to investigate the role of CXCL14 in GBM malignancy, we first treated cells with exogenous CXCL14 (400 ng/mL) [42] and observed significant enhancement of cell migration (U87: *p* < 0.01; U251: *p* < 0.01; T98G: *p* < 0.01) (Figures 3B,C and S4E). Transwell assays further confirmed increased invasive and migratory capacities (U87: *p* < 0.001; U251: *p* < 0.001; T98G: *p* < 0.001) (Figure 3D,E), while WB analysis revealed alterations in EMT‐related proteins (All comparisons *p* < 0.05; see Table S3 for complete statistical details) (Figure 3F–I). Using lentiviral vectors carrying CXCL14 shRNA or overexpression plasmids, we successfully established cell lines with either reduced or elevated CXCL14 expressions. ELISA and qRT‐PCR results verified the transfection efficiency and identified two optimal clones with the most effective CXCL14 knockdown (All comparisons *p* < 0.05; see Table S3 for complete statistical details) (Figure S4A–D) (Primer sequences used can be found in Table S1). Subsequent scratch wound healing assays demonstrated that CXCL14 silencing significantly inhibited cell migration, whereas CXCL14 overexpression enhanced migratory capacity (All comparisons *p* < 0.05; see Table S3 for complete statistical details) (Figures 3J–M and S4F,G). Further transwell experiments showed that CXCL14‐knockdown groups exhibited fewer migrating and invading cells compared to controls, while overexpression groups displayed increased numbers of migrating and invading cells (All comparisons *p* < 0.05; see Table S3 for complete statistical details) (Figure 3N–Q). WB analysis demonstrated significant alterations in EMT‐related proteins following both CXCL14 knockdown and overexpression (All comparisons *p* < 0.05; see Table S3 for complete statistical details) (Figure 3R–Y). These findings suggested that CXCL14 promoted migration, invasion, and EMT in GBM cells.

### 3.4 c‐FOS was a Potential Transcription Factor of CXCL14

**FIGURE 4:**
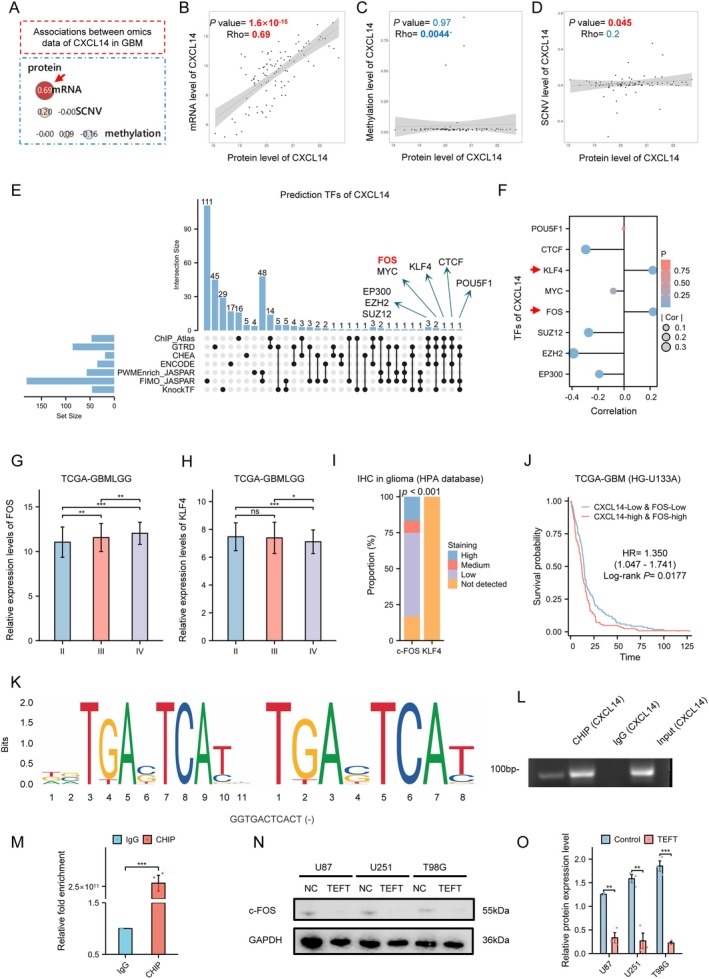
c‐FOS is a potential transcription factor of CXCL14. (A) Associations between omics data of CXCL14 in GBM. Spearman *r*‐test. (B) Correlation analysis between protein and mRNA levels of CXCL14 in GBM. Spearman *r*‐test. (C) Correlation analysis between protein and methylation levels of CXCL14 in GBM. Spearman *r*‐test. (D) Correlation analysis between protein and SCNV levels of CXCL14 in GBM. Spearman *r*‐test. (E) Upset plot screening potential TFs of CXCL14. (F) Dot plot analyzing potential TFs positively correlated with CXCL14. Spearman *r*‐test. (G) Expression profile of FOS across II, III and IV grades glioma. Data are mean ± SD, Kruskal‐Wallis test, and Dunn's post hoc test. **p < 0.01, ***p < 0.001. (H) Expression profile of KLF4 across II, III and IV grades glioma. Data are mean ± SD, Kruskal‐Wallis test, and Dunn's post hoc test. ns, *p* ≥ 0.05, *p < 0.05, ***p < 0.001. (I) Comparison c‐FOS and KLF4 IHC staining results. Fisher's exact test. (J) Survival analysis of TCGA‐GBM patients (CXCL14‐high and FOS‐high vs. CXCL14‐low and FOS‐low), Log‐rank test. (K) Conserved sequence logo of the c‐FOS transcription factor binding site predicted by the JASPAR database, along with the highest‐scoring predicted binding site. (L–M) ChIP‐PCR and ChIP‐qPCR experiments qualitatively and quantitatively confirmed that c‐FOS functions as a direct transcriptional regulator of CXCL14 (***p < 0.001). (N, O) WB analysis revealed a significant reduction in c‐FOS protein levels following TEFT (U87: **p < 0.01, U251: **p < 0.01, T98G: ***p < 0.001).

Associations analyses suggested that CXCL14 protein had the highest correlation with CXCL14 mRNA (Figure 4A,B), compared to CXCL14 methylation levels (Figure 4C) and CXCL14 SCNV levels (Figure 4D) (Correlation between CXCL14 protein and mRNA: Rho = 0.22, *p* = 1.60 × 10^−15^; Correlation between CXCL14 protein and methylation: Rho = 4.40 × 10^−3^, *p* = 0.97; Correlation between CXCL14 protein and SCNV: Rho = 0.20, *p* = 0.045). The above results suggested that transcriptional regulation of CXCL14 was more important in GBM. By integrating transcription factor binding information from multiple databases (ChIP Atlas, GTRD, CHEA, ENCODE, PWMEnrich JASPAR, FIMO JASPAR and KnockTF), we screened for potential transcription factors associated with CXCL14. Eight transcription factors showed consensus across four databases (FOS, MYC, EP300, EZH2, SUZ12, KLF4, CTCF, POU5F1) (Figure 4E). Among these, KLF4 and FOS demonstrated significant positive correlation with CXCL14 expression (KLF4: Rho = 0.22, *p* = 4.28 × 10^−3^; FOS: Rho = 0.22, *p* = 3.82 × 10^−3^; EP300: Rho = −0.19, *p* = 1.39 × 10^−2^; EZH2: Rho = −0.39, *p* = 2.18 × 10^−7^; SUZ12: Rho = −0.27, *p* = 3.87 × 10^−4^; MYC: Rho = −0.08, *p* = 0.29; CTCF: Rho = −0.29, *p* = 1.20 × 10^−4^; POU5F1: Rho = −2.71 × 10^−3^, *p* = 0.97) (Figure 4F). While FOS expressions increased with glioma grade progression (Figure 4G), KLF4 showed no significant differential expression between grade II and III gliomas (Figure 4H). IHC staining results presented undetectable levels of KLF4 protein in comparison with c‐FOS (2 high staining, 1 medium staining and 7 low staining vs. 14 not detected staining) (Figure 4I). Survival analysis indicated poor prognosis of GBM patients with high expression FOS and CXCL14 (HR = 1.350, 95% CI: 1.047–1.741; Log‐rank *p* = 0.0177) (Figure 4J). Using JASPAR to identify the c‐FOS motif sequence and Ensembl to obtain the CXCL14 promoter sequence, we identified the highest‐scoring predicted binding sequence (GGTGACTCACT) (Figure 4K). Subsequent ChIP‐PCR and ChIP‐qPCR experiments qualitatively and quantitatively confirmed that c‐FOS functions as a direct transcriptional regulator of CXCL14 (*p* < 0.001) (Figure 4L,M) (Primers used for ChIP assays are listed in Table S1). Meanwhile, we found that the c‐FOS protein was significantly reduced in TEFT‐exposed cells (U87: *p* < 0.01; U251: *p* < 0.01; T98G: *p* < 0.001) (Figure 4N,O). In conclusion, c‐FOS may serve as a potential transcriptional regulator of CXCL14.

### 3.5 c‐FOS Promoted Migration, Invasion, and EMT in GBM Through CXCL14

**FIGURE 5:**
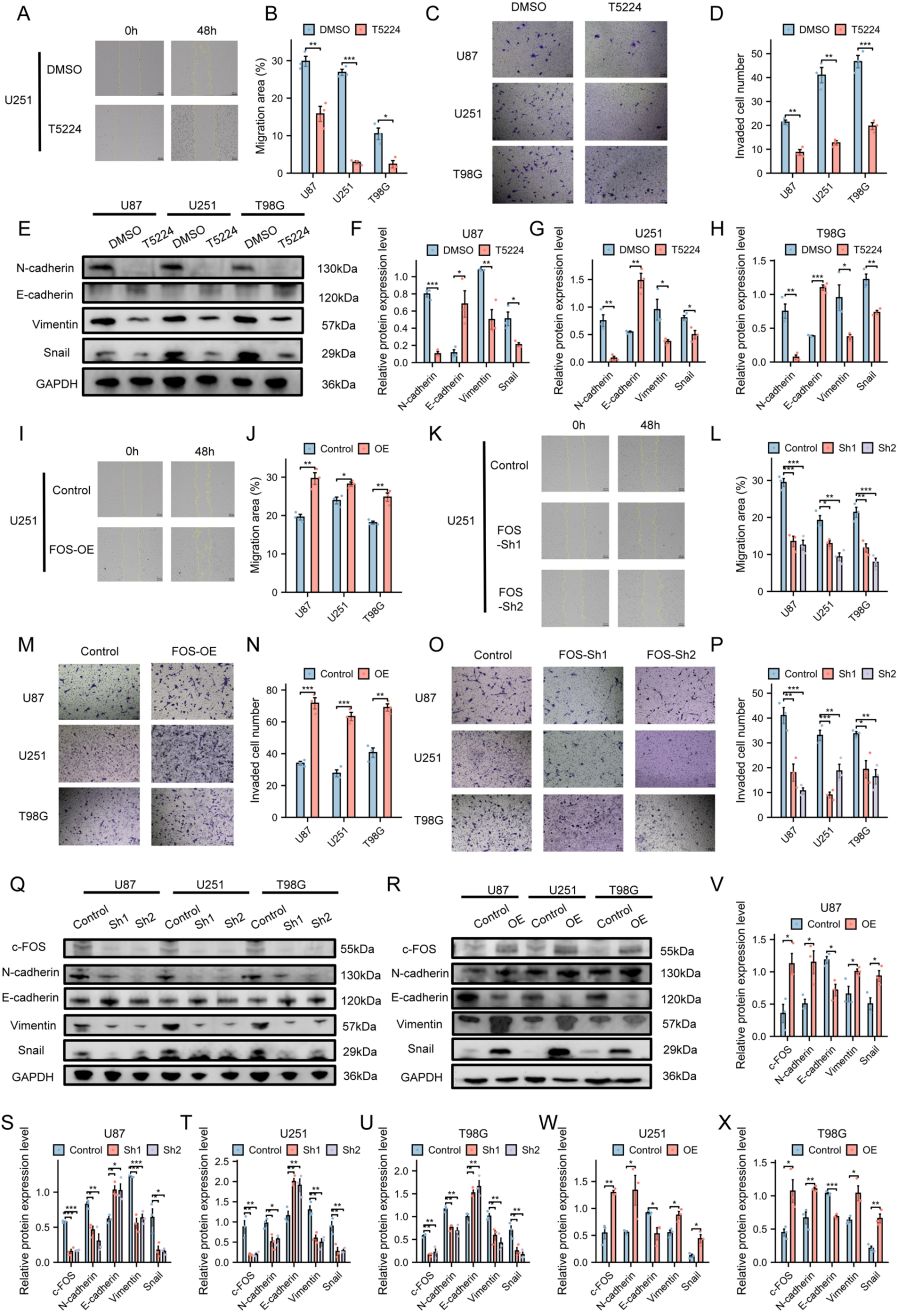
c‐FOS promoted migration, invasion, and EMT in GBM. (A, B) Wound healing assay of U251 cells following c‐FOS inhibitor T‐5224 treatment and statistical results across three cell lines, Student's *t*‐test, *n* = 3, *p < 0.05, **p < 0.01, ***p < 0.001. Scale bar = 200 μm. (C, D) Invasion assay and statistical results of three cell lines following T‐5224 treatment, Student's *t*‐test, *n* = 3, *p < 0.05, **p < 0.01, ***p < 0.001. Scale bar = 100 μm. (E–H) WB analysis and statistical results of three cell lines following T‐5224 treatment, Student's *t*‐test, *n* = 3, *p < 0.05, **p < 0.01, ***p < 0.001. (I–K) Wound healing assay in U251 cells following FOS overexpression and knockdown with statistical analysis across three cell lines, Student's *t*‐test for two groups, one‐way ANOVA with Tukey's post hoc test for three groups, *n* = 3, *p < 0.05, **p < 0.01, ***p < 0.001. Scale bar = 200 μm. (M–P) Invasion assay and statistical analysis of three cell lines following FOS overexpression and knockdown, Student's *t*‐test for two groups, one‐way ANOVA with Tukey's post hoc test for three groups, *n* = 3, *p < 0.05, **p < 0.01, ***p < 0.001. Scale bar = 100 μm. (Q–X) WB analysis and statistical analysis of three cell lines following FOS overexpression and knockdown, Student's *t*‐test for two groups, one‐way ANOVA with Tukey's post hoc test for three groups, *n* = 3, *p < 0.05, **p < 0.01, ***p < 0.001.

To demonstrate that c‐FOS is a transcription factor for CXCL14, treatment with T‐5224, a c‐FOS inhibitor, markedly attenuated cell migration (U87: *p* < 0.01; U251: *p* < 0.001; T98G: *p* < 0.05) (Figures 5A,B and S5E). Transwell assays further confirmed decreased invasive capacity (U87: *p* < 0.01; U251: *p* < 0.01; T98G: *p* < 0.001) (Figure 5C,D), while WB analysis revealed alterations in EMT‐related protein expression (All comparisons *p* < 0.05; see Table S3 for complete statistical details) (Figure 5E–H). Using lentiviral vectors carrying FOS shRNA or overexpression plasmids, we successfully established stable cell lines with either reduced or elevated FOS expression. qRT‐PCR and WB results verified transfection efficiency and identified two optimal clones with the most effective FOS knockdown (Primer sequences used can be found in Table S1). Subsequent ELISA experiments detected that alterations in FOS expression levels were positively correlated with changes in CXCL14 levels in the supernatant (All comparisons *p* < 0.05; see Table S3 for complete statistical details) (Figures 5Q,R and S5A–D). Subsequent scratch wound healing assays demonstrated that FOS silencing significantly inhibited cell migration, whereas FOS overexpression enhanced migratory capacity (All comparisons *p* < 0.05; see Table S3 for complete statistical details) (Figure 5I–L and S5F,G). Transwell assays showed that the FOS‐knockdown groups had fewer invading cells compared to controls, while the overexpression groups exhibited a significant increase in invading cells (All comparisons *p* < 0.05; see Table S3 for complete statistical details) (Figure 5M–P). WB analysis indicated that both FOS knockdown and overexpression induced notable changes in EMT‐related proteins (All comparisons *p* < 0.05; see Table S3 for complete statistical details) (Figure 5Q–X). To confirm the hierarchical relationship, we supplemented exogenous CXCL14 in FOS‐knockdown cell lines and repeated the above experiments (All comparisons *p* < 0.05; see Table S3 for complete statistical details) (Figure S6A–J). The results showed that cells regained mesenchymal characteristics, suggesting that FOS may promote migration, invasion, and EMT in GBM cells through CXCL14.

### 3.6 TEFT Promoted Proteasomal Degradation of c‐FOS

We observed that WB analysis indicated significant alterations in c‐FOS protein levels following TEFT treatment. Literature review revealed that early experimental studies had already documented the inherent instability of FOS mRNA, potentially determined by multiple cis‐acting elements [43, 44]. Subsequent research identified post‐transcriptional regulatory proteins that maintain their mRNA stability [44, 45, 46], while studies over the past decade had increasingly highlighted miRNA‐mediated regulation of its expression [47, 48]. Therefore, we hypothesized that TEFT may exert its effects by influencing FOS mRNA translation, through potential mechanisms such as modulating mRNA stability [49], regulating ribosomal translation speed [50], or altering protein conformation [51]. However, RNA stability assays contradicted our hypothesis by revealing a temporary compensatory rise in FOS mRNA following TEFT stimulation (All comparisons *p* < 0.05) (Figure S6K–M). Because the c‐FOS protein is structurally highly labile and its rapid turnover is strictly governed by cellular degradation machinery [52], we redirected our investigation toward posttranslational mechanisms. To determine whether the c‐FOS reduction driven by TEFT operates via the ubiquitin proteasome system, we utilized the proteasome inhibitor MG132. Subsequent immunoblotting revealed that proteasomal inhibition significantly attenuated the c‐FOS downregulation initiated by TEFT (All comparisons *p* < 0.05) (Figure S6N,O). Collectively, these findings offer compelling evidence that TEFT actively promoted c‐FOS depletion through a process entirely reliant on proteasomal degradation, rather than repressing its transcription or translation.

### 3.7 In Vivo Studies Validate the Inhibitory Effect of TEFT on c‐FOS/CXCL14 Axis and EMT

**FIGURE 6:**
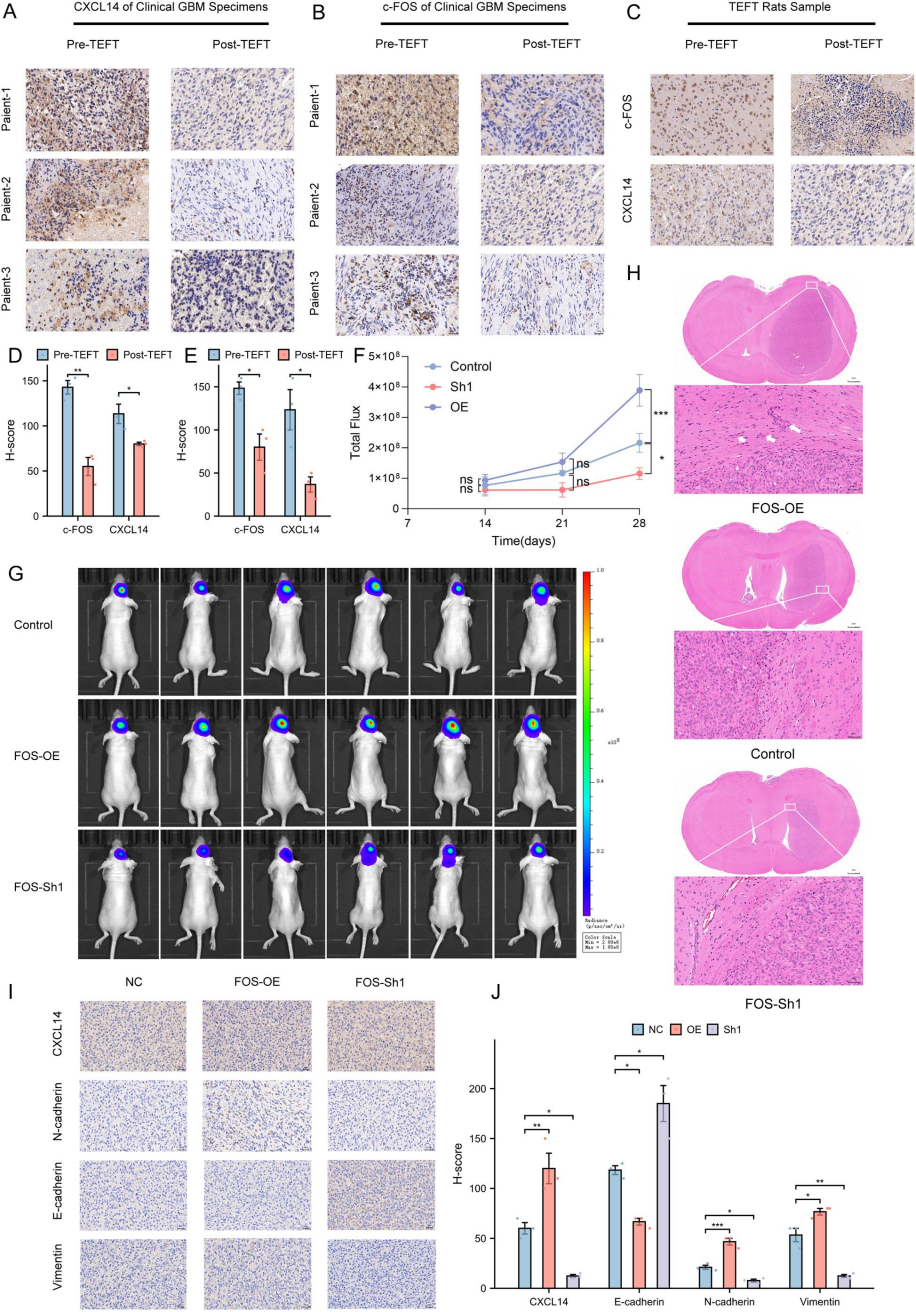
In vivo studies validate the inhibitory effect of TEFT on the c‐FOS/CXCL14 axis and EMT. (A, B, D) IHC results and H‐score assessment of c‐FOS and CXCL14 in GBM patients' tissues following TEFT, Student's *t*‐test, *n* = 3, *p < 0.05, **p < 0.01, ***p < 0.001. Scale bar = 30 μm. (C, E) IHC results and H‐score assessment of c‐FOS and CXCL14 in GBM‐bearing rats before and after TEFT. Scale bar = 30 μm. (F, G) In vivo imaging of nude mice with orthotopic implantation of U251 cell lines: NC, FOS‐knockdown, and FOS‐overexpressing models; fluorescence intensity at days 14, 21, and 28; and representative images at day 28. (OE: ***p < 0.001, Sh1: *p < 0.05, two‐way ANOVA with Tukey's post hoc test for three groups, *n* = 6) (H) HE staining revealed prominent tissue invasion and scattered metastatic tumor islands in the OE group compared to the other two groups. Scale bar = 30 μm. (I, J) IHC results and statistical analysis of N‐cadherin, E‐cadherin, and Vimentin in brain tissues of GBM‐bearing mice, one‐way ANOVA with Tukey's post hoc test for three groups, *n* = 3, *p < 0.05, **p < 0.01, ***p < 0.001. Scale bar = 30 μm.

We further validated the inhibitory effect of TEFT on the malignant progression of GBM using clinical specimens and animal models. IHC analysis of tissues from three GBM patients who underwent TEFT treatment revealed significantly reduced expression of both CXCL14 and c‐FOS post‐treatment (c‐FOS: *p* < 0.01, CXCL14: *p* < 0.05) (Figure 6A,B,D). Similarly, IHC examination of rat brain GBM samples demonstrated that TEFT treatment markedly downregulated the expression levels of CXCL14 and c‐FOS, suggesting that TEFT effectively suppresses the c‐FOS/CXCL14 axis (c‐FOS: *p* < 0.05, CXCL14: *p* < 0.05) (Figure 6C,E). Subsequently, we intracranially implanted athymic mice with U251 wild‐type cells, along with FOS‐knockdown and FOS‐overexpressing cells, all modified to express firefly luciferase via lentiviral transduction. Two weeks post‐transplantation, tumor formation was confirmed by in vivo bioluminescence imaging. In vivo imaging sessions were performed to monitor tumor size progression weekly. On day 28, significant differences became apparent among the groups (Sh1: *p* < 0.01 OE: *p* < 0.001) (Figure 6F,G). Brain tissues were then harvested and subjected to HE staining, which revealed that the FOS‐overexpression group exhibited markedly enhanced tissue infiltration and the presence of invasive tumor satellite islands compared to the other groups (Figure 6H). IHC analysis of the tumor tissues demonstrated substantial alterations in the downstream molecule CXCL14 and EMT markers (N‐cadherin, E‐cadherin, Vimentin) (All comparisons *p* < 0.05; see Table S3 for complete statistical details) (Figure 6I,J). These experimental findings collectively demonstrate that FOS plays a critical role in promoting glioblastoma invasion and metastasis through regulation of the CXCL14‐mediated EMT pathway in vivo.

## 4 Discussion

TEFT represents a groundbreaking advancement in GBM therapy, demonstrating significant clinical efficacy [49, 53]. Particularly in both newly diagnosed and recurrent GBM, TEFT prolongs OS and progression‐free survival with minimal adverse effects, earning the designation as the “fourth modality of cancer treatment.” However, the precise molecular mechanisms underlying its antitumor effects remain incompletely elucidated. This study reveals for the first time that chemokine CXCL14 is a key mediator of TEFT in GBM treatment. The mechanism involves TEFT‐mediated suppression of c‐FOS, leading to downregulation of CXCL14 expression, subsequent inhibition of EMT, and reduction of the aggressive mesenchymal phenotype in GBM. These findings deepen our understanding of TEFT mode of action and provide new insights for improving GBM treatment strategies.

EMT is a critical biological process wherein cells lose polarity and adhesion properties while acquiring enhanced migratory and invasive mesenchymal characteristics [54]. EMT represents a reversible cellular state [55] and is categorized into three distinct subtypes: Type 1 EMT participates in embryogenesis and organ development; Type 2 EMT is associated with wound healing, tissue regeneration, and organ fibrosis [56]; and Type 3 EMT in GBM arises from genetically and epigenetically altered neoplastic cells responsible for primary tumor development [57]. As GBM cells approach a mesenchymal state, they gain increased capacity to migrate and invade blood vessels and basement membranes. Since GBM cells do not originate from epithelial tissues, the EMT process in GBM should be referred to as EMT‐like [58]. Clinically, tumor recurrence after resection often results from highly infiltrative cells penetrating the brain, with enhanced cell infiltration driven by multiple processes, among which EMT‐related signaling pathways play a crucial role [59]. Furthermore, the scope of programmed biological processes in tumorigenesis extends far beyond cancer cell invasion. Activation of the EMT program is closely associated with tumor microenvironment (TME) regulation, transition into cancer stem cell states, and acquisition of therapy resistance [60]. Clinically, EMT and Proneural‐to‐Mesenchymal Transition (PMT) are pivotal mechanisms underlying therapeutic resistance and heightened invasiveness in GBM [61, 62]. In recurrent GBM, MES subtype emerges as the dominant phenotype [63], while the proportions of PN and CL subtypes, which are prevalent at initial diagnosis, decrease significantly. This shift suggests that PMT is a core driver of tumor progression and recurrence [64]. The ability of TEFT to effectively block both EMT and PMT processes indicates that this therapy may hold particular therapeutic promise for patients with MES subtype GBM. Therefore, our study on TEFT‐mediated suppression of EMT holds significant importance in GBM therapy.

CXCL14, a structurally conserved chemokine constitutively expressed in epithelial cells and fibroblasts [65], is closely linked to EMT and glioma progression. CXCL14 has been demonstrated to significantly correlate with EMT in multiple tumors [66, 67, 68]. In GBM, CXCL14 promotes tumorigenicity and invasiveness by binding to IGF‐1R and activating downstream signaling pathways [69]. Additionally, CXCL14 facilitates GBM progression by modulating the immune microenvironment [70], though its specific mechanisms are multidimensional and complex. By establishing more “epithelialized” and “mesenchymalized” GBM cell models, we identified CXCL14 as a DEGs through transcriptomic analysis and validated it using public databases. Experimental evidence confirmed CXCL14 as a key molecule driving EMT in GBM cells, while TEFT likely suppresses EMT in GBM by reducing CXCL14 expression. Although CXCL14 has been considered an orphan chemokine without a definitively identified receptor, recent studies have proposed potential receptors such as MRGPRX2, CXCR4, and IGF‐1R [69, 71, 72], which may play auxiliary or synergistic roles in specific tissues or pathological contexts. However, conflicting experimental observations from different studies have led to discrepancies in understanding CXCL14's functions [42, 65], warranting further investigation into its downstream receptors and signaling pathways. c‐FOS, a product of the proto‐oncogene FOS, holds significant roles in the nervous system, including learning and memory [73], control of central nervous system tumor growth [74], and notable neuroprotection [75]. The role of c‐FOS in EMT remains controversial: some studies suggest it may positively regulate EMT in certain tissues [28, 76], while others indicate that its upregulation suppresses the mesenchymal phenotype in different contexts [29, 77].

In our experiments, multi‐database integration revealed the relationship between c‐FOS and CXCL14 in GBM. CHIP‐PCR and CHIP‐qPCR qualitatively and quantitatively confirmed their binding. We further demonstrated that TEFT suppresses GBM cells' EMT both in vivo and in vitro via the c‐FOS/CXCL14 axis, providing a novel theoretical foundation for TEFT in GBM therapy. Building upon the team's previous research, COL6A1 constitutes the foundational physical matrix that supports tumor cell migration [10], while CXCL14 provides the biochemical signals driving EMT and invasive progression. Through the dual inhibition of these two targets, TEFT could effectively dismantle the physical scaffold and biochemical signaling networks upon which mesenchymal GBM relies for survival. TME is a complex ecosystem composed of tumor cells and their surrounding cellular, molecular, and stromal components, which profoundly impacts tumor growth, metastasis, and drug resistance [78]. Within this context, the c‐FOS/CXCL14 axis and EMT play crucial roles. Cancer‐associated fibroblasts (CAFs) serve as vital sources and regulators of CXCL14, enabling it to promote cell invasion and migration [79]. Concurrently, studies have found that exosomal CXCL14 secreted by prostate cancer cells induces macrophage polarization toward the M2 phenotype via the NF‐κB signaling pathway, thereby indirectly facilitating tumor migration and invasion [80]. The significance of targeting the c‐FOS/CXCL14 axis extends beyond merely suppressing the intrinsic invasive machinery of tumor cells. More crucially, it holds the potential to disrupt the paracrine communication network through which tumor cells utilize CXCL14 to reprogram the microenvironment. Consequently, it is highly plausible that TEFT could intervene in tumor progression by modulating these specific pathways, which warrants further investigation.

Therefore, we believe that TEFT holds promising clinical potential by suppressing the c‐FOS/CXCL14 axis, thereby inhibiting EMT in GBM. Through complex regulatory networks, its applications can be further explored across multiple dimensions, including TME and immunity, particularly concerning clinical MES subtypes, recurrent GBM, and combination immunotherapy.

This study has several limitations that should be addressed in future research. First, the high heterogeneity of GBM implies significant variations among tumor subtypes and individual patients, which may impact the general applicability of the c‐FOS/CXCL14 axis in TEFT therapy. Additionally, while the c‐FOS/CXCL14 axis represents an upstream regulatory pathway in TEFT, the mechanisms by which TEFT influences c‐FOS protein expression and the downstream receptors and signaling pathways of CXCL14 remain incompletely elucidated. Moreover, the regulatory roles of c‐FOS and CXCL14 in tumor mechanisms are exceedingly complex, potentially involving tumor immune regulation. Furthermore, a TMZ group was not included as a positive control in our in vivo experiments. As a result, we were unable to further explore the significant potential of TEFT‐mediated EMT inhibition in overcoming chemoresistance and enhancing comprehensive clinical therapeutic efficacy. This limitation should be carefully addressed in future research. The use of nude mice may not fully recapitulate the complex intracranial microenvironment of GBM. Future studies should employ models that better reflect GBM biology to identify clearer mechanisms and pathways, thereby enabling the development of more precise TEFT‐based therapeutic strategies. In contrast to traditional cell line‐derived xenograft models, patient‐derived xenograft models preserve the heterogeneity and tumor microenvironment including partial vascular and stromal components of the original patient tumors, and are thus considered more clinically relevant preclinical models. Such models have been widely adopted in GBM research [81, 82, 83]. This represents a key direction for potential improvement in our subsequent studies.

## Author Contributions

C.S. wrote the manuscript, C.S., Y.L. and J.C. conducted bioinformatics data analysis and acquired experiment data. All authors discussed the results. J.P., C.L., J.L., and E.W. contributed to validation and revised the manuscript. J.L., Z.L. and L.C. reviewed, supervised and acquired support. All authors read and approved of the final manuscript.

## Funding

This work was supported by the National Natural Science Foundation of China (82172680, 82373220, 82473264, and 82403942).

## Ethics Statement

The study was approved by the Ethics Committee of PLA General Hospital (Batch No. S2018‐089‐01).

## Consent

The authors have nothing to report.

## Conflicts of Interest

The authors declare no conflicts of interest.

Ling Chen is an Academic Editor of CNS Neuroscience and Therapeutics and a co‐author of this article. To minimize bias, they were excluded from all editorial decision‐making related to the acceptance of this article for publication.

## Supporting information


**Figure S1:** CXCL14 expression heterogeneity in Glioma databases.
**Figure S2:** The MES subtype may exhibit a significant association with the EMT process.
**Figure S3:** Validation of crucial role of CXCL14 in EMT process.
**Figure S4:** Alterations in cell lines following CXCL14 expression modulation.
**Figure S5:** Alterations in cell lines following c‐FOS expression modulation.
**Figure S6:** c‐FOS acts as an upstream regulator of CXCL14.
**Table S1:** The primer sequences used for qRT‐PCR and ChIP assays.
**Table S2:** The antibodies used in this study.
**Table S3:** The detailed *p*‐values.

**Supporting Information Methods.** A comprehensive description of all experimental procedures, materials, and analytical methods is provided in the Supplementary Methods document.

## Data Availability

The datasets used and analyzed during the current study are available from the corresponding author on reasonable request.
