# Supplementary material for: Tumor Electric Field Therapy Inhibits Epithelial‐Mesenchymal Transition, Invasion, and Migration of Glioblastoma by Targeting the c‐FOS/CXCL14 Axis
Source: CNS Neurosci Ther. 2026 May 19;32(5):e70926. doi: 10.1002/cns.70926 (PMC13185563; doi:10.1002/cns.70926)
Supplement: Supplementary file 1 — Figure S1: CXCL14 expression heterogeneity in Glioma databases. Figure S2: The MES subtype may exhibit a significant association with the EMT process. Figure S3: Validation of crucial role of CXCL14 in EMT process. Figure S4: Alterations in cell lines following CXCL14 expression modulation. Figure S5: Alterations in cell lines following c‐FOS expression modulation. Figure S6: c‐FOS acts as an upstream regulator of CXCL14. Table S1: The primer sequences used for qRT‐PCR and ChIP assays. Table S2: The antibodies used in this study. Table S3: The detailed p‐values. [file CNS-32-e70926-s001.docx]

**
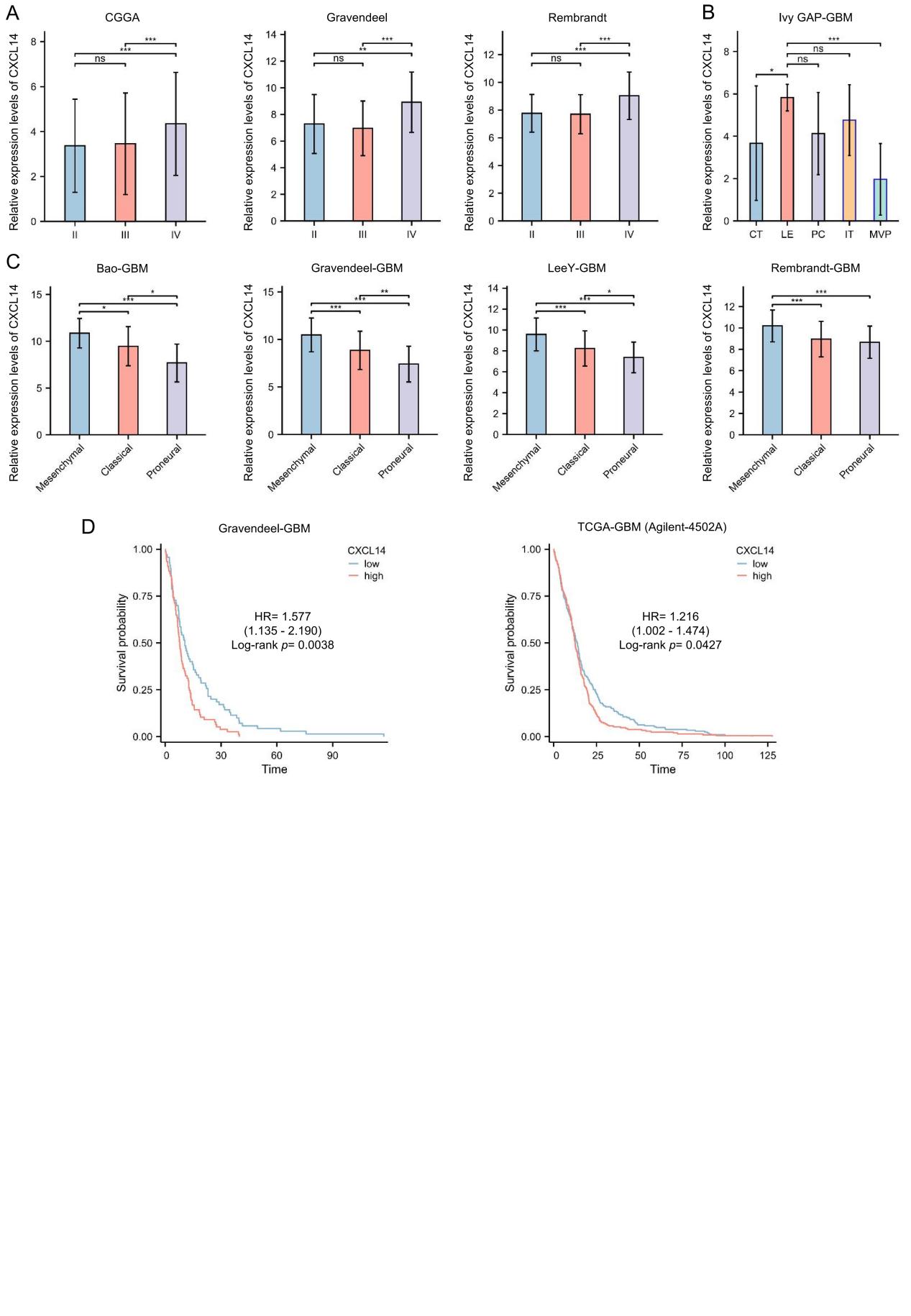
**

**Figure S1 CXCL14 expression heterogeneity in Glioma databases**

A. CXCL14 expression in WHO grade IV gliomas is significantly elevated compared to grade III and lower-grade tumors across three datasets (CGGA, Gravendeel, and Rembrandt). (CGGA ****p* < 0.001, Gravendeel ****p* < 0.001, Rembrandt ****p* < 0.001).

B. In the Ivy GAP-GBM dataset, heterogeneous CXCL14 expression is observed across distinct anatomical regions, with microvascular proliferative zones and cellular tumor regions exhibiting significantly higher expression levels compared to the Lesion Edge. (MVP vs. LE ****p* < 0.001, CT vs. LE **p* < 0.05)

C. CXCL14 expression is significantly elevated in the mesenchymal subtype compared to other molecular subtypes across four GBM datasets (Bao, Gravendeel, LeeY, and Rembrandt), one-way ANOVA with Tukey’s post hoc test for three groups, n=3, **p* < 0.05, ***p* < 0.01, ****p* < 0.001.

D. Survival analysis plots comparing high and low CXCL14 expression groups in two GBM databases (Gravendeel, TCGA). (Gravendeel-GBM: HR= 1.577 (1.135 - 2.190) Log-rank *p*= 0.0038, TCGA-GBM: HR= 1.216 (1.002 - 1.474) Log-rank *p* = 0.0427)

**
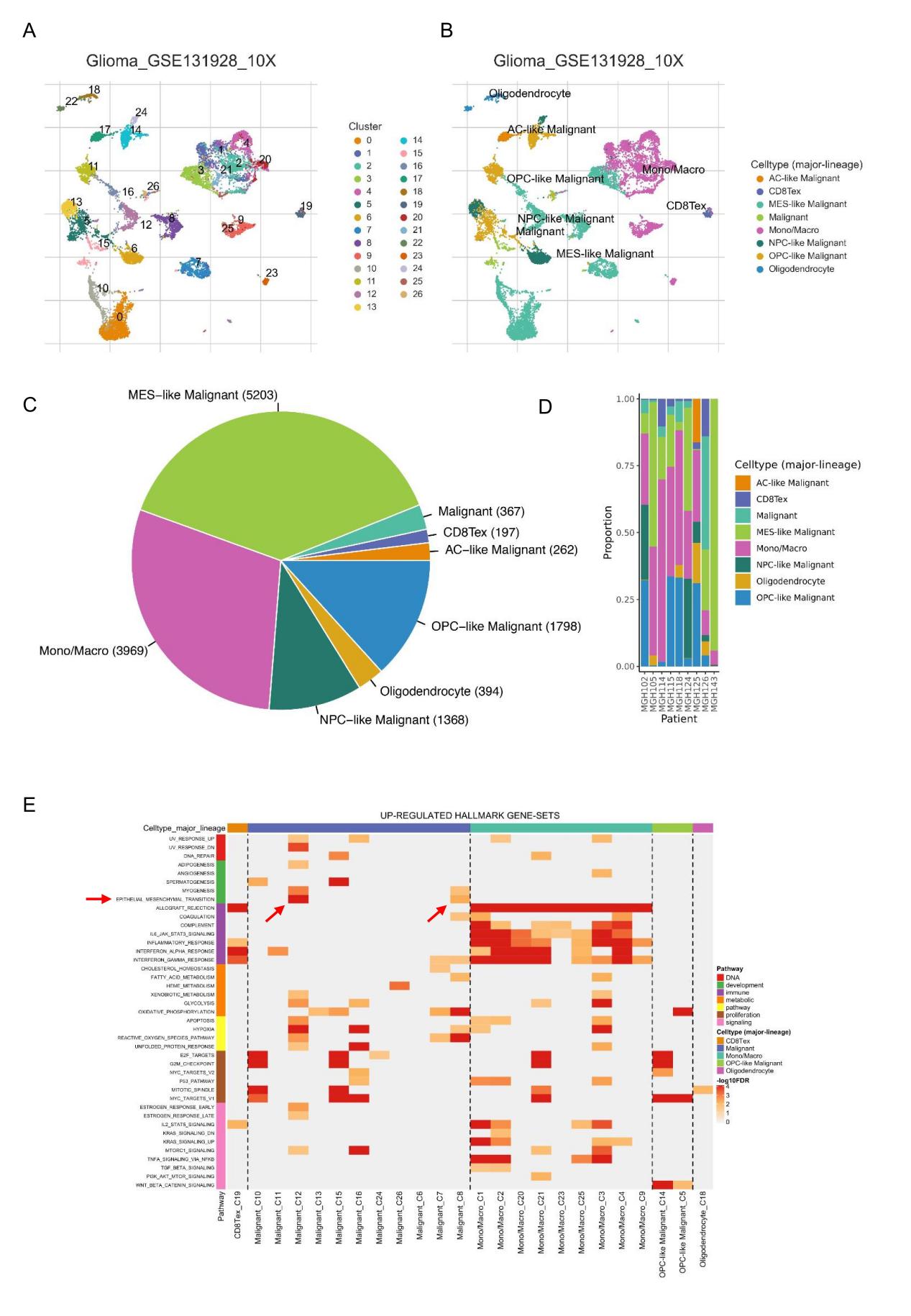
**

**Figure S2 The MES subtype may exhibit a significant association with the EMT process.**

A-B. Single-cell RNA sequencing data reveal partial overlaps among CXCL14-high subpopulations, EMT-high clusters, and mesenchymal subtype clusters.

C. Single-cell profiling reveals the cellular heterogeneity in glioblastoma (GBM), with MES-like Malignant cells (5,203) representing the predominant subpopulation, underscoring the predominance of the mesenchymal subtype in GBM.

D. The mesenchymal subtype represents a clinically significant subgroup in glioma patients, highlighting the therapeutic relevance of targeting EMT pathways in GBM.

E. GSEA heatmap demonstrates significant enrichment of the EMT pathway in Malignant_C12 and Malignant_C8 subpopulations, which are identified as mesenchymal subtype clusters.


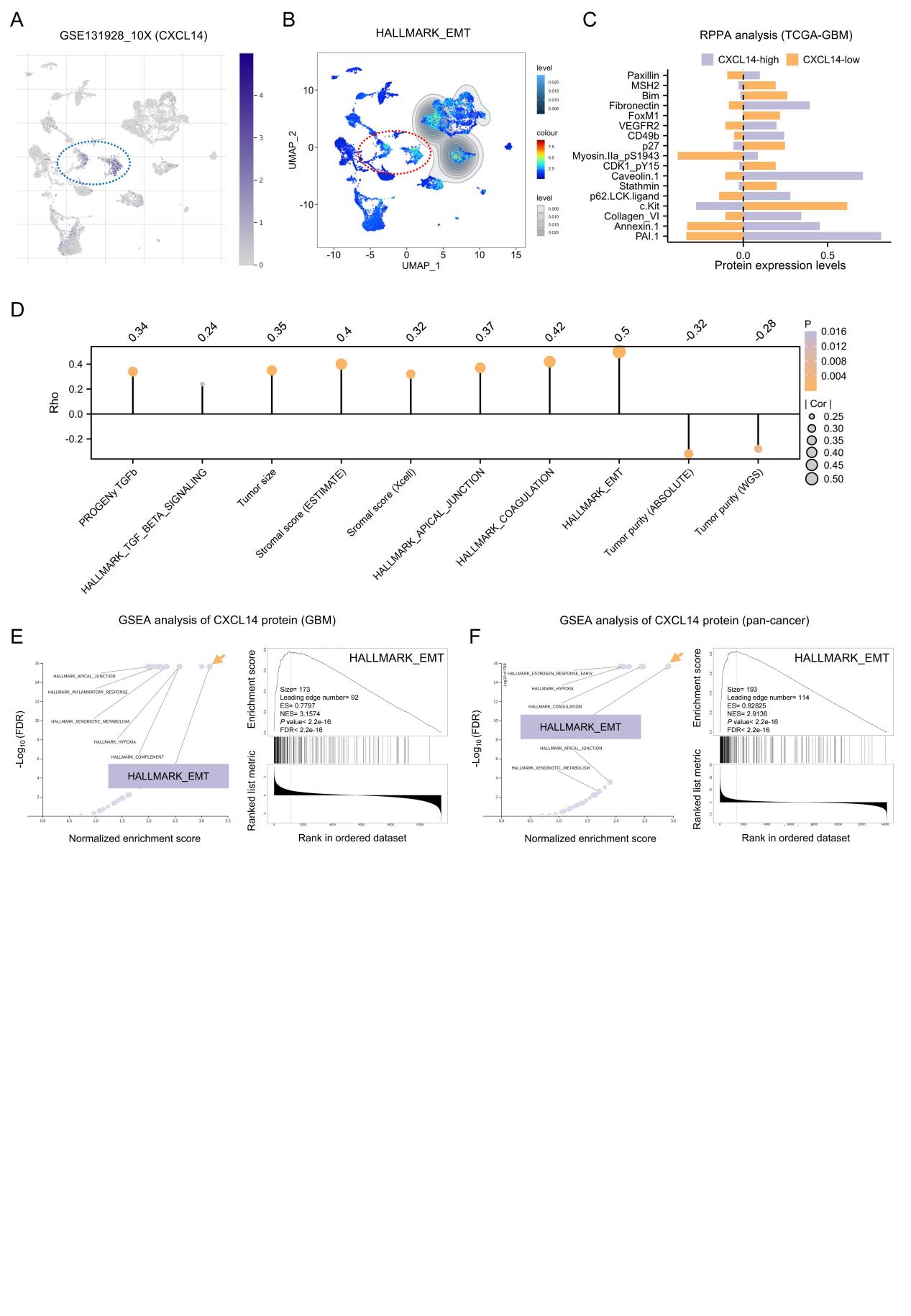


**Figure S3 Validation of crucial role of CXCL14 in EMT process**

A. UMAP plot depicted CXCL14 expression levels across different cell types.

B. UMAP plot depicted EMT levels across different cell types.

C. RPPA analysis between CXCL14-high and CXCL14-low GBM.

D. Dot plots illustrated correlation results between CXCL14 protein and crucial phenotypes. Spearman *r*-test.

E-F. CXCL14 protein related to GSEA analyses on GBM and pan-cancer level.

**
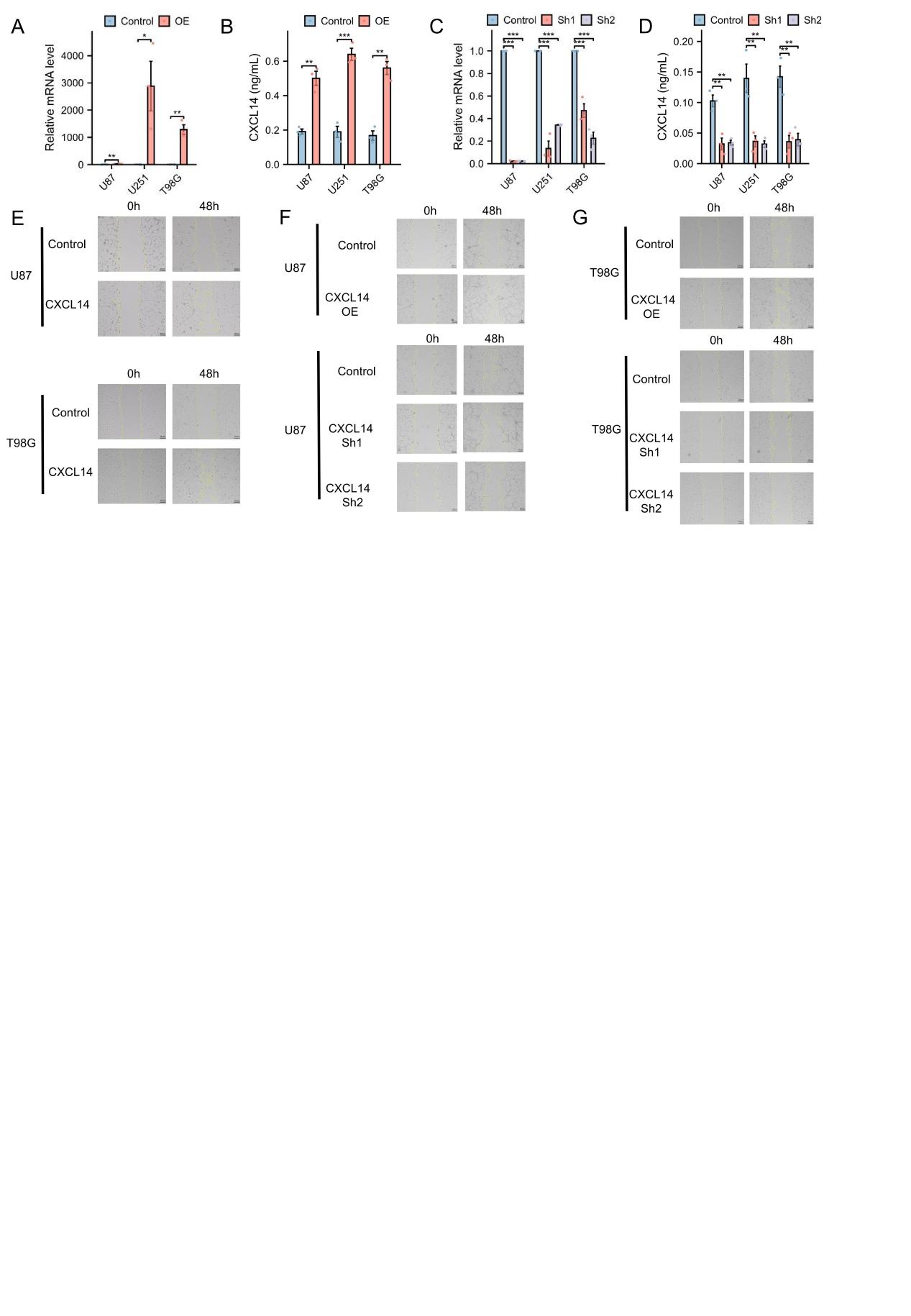
**

**Figure S4 Alterations in cell lines following CXCL14 expression modulation**

A. qRT-PCR validation of overexpression efficiency across cell lines. (U87 ***p* < 0.01, U251 **p* < 0.05, T98G ***p* < 0.01).

B. ELISA detection of CXCL14 levels following overexpression. (U87 ***p* < 0.01, U251 ****p* < 0.001, T98G ***p* < 0.01).

C. Assessment of knockdown efficiency across cell lines via qRT-PCR (U87Sh1 ****p* < 0.001, U87Sh2 ****p* < 0.001; U251Sh1 ****p* < 0.001, U251Sh2 ****p* < 0.001; T98GSh1 ****p* < 0.001, T98GSh2 ****p* < 0.001).

D. ELISA detection of CXCL14 levels following knockdown (U87Sh1 ****p* < 0.001, U87Sh2 ****p* < 0.001; U251Sh1 ****p* < 0.001, U251Sh2 ****p* < 0.001; T98GSh1 ****p* < 0.001, T98GSh2 ****p* < 0.001).

E. Wound healing assay in U87 and T98G cells following exogenous CXCL14 supplementation. Scale bar= 200μm.

F, G. Wound healing assay in U87 and T98G cells following CXCL14 overexpression and knockdown. Scale bar= 200μm.

**
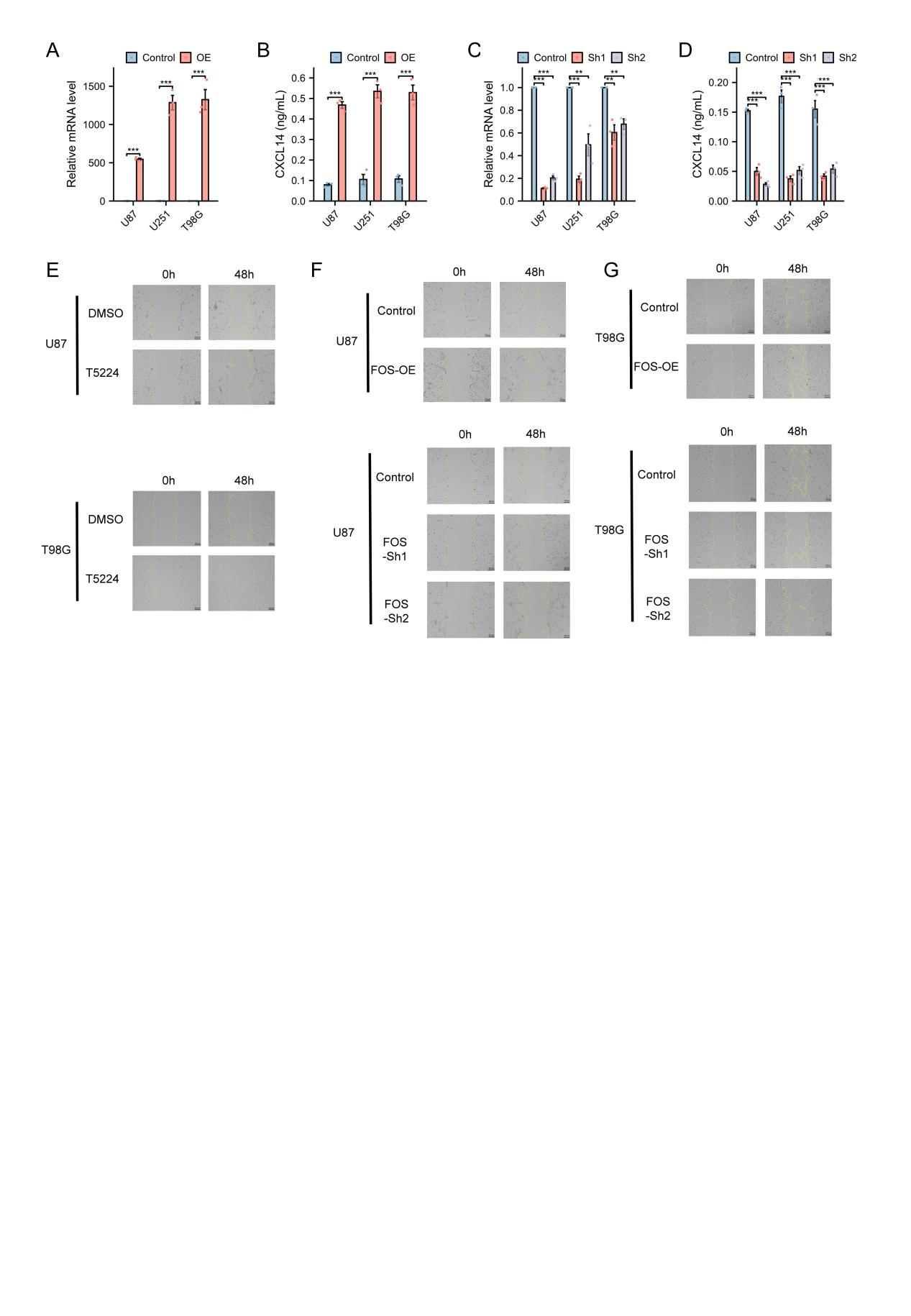
**

**Figure S5 Alterations in cell lines following c-FOS expression modulation**

A. qRT-PCR validation of overexpression efficiency across cell lines (U87 ****p* < 0.001; U251 ****p* < 0.001; T98G ****p* < 0.001).

B. ELISA detection of CXCL14 levels following overexpression (U87 ****p* < 0.001; U251 ****p* < 0.001; T98G ****p* < 0.001).

C. Assessment of knockdown efficiency across cell lines via qRT-PCR (U87Sh1 ****p* < 0.001, U87Sh2 ****p* < 0.001; U251Sh1 ****p* < 0.001, U251Sh2 ***p* < 0.01; T98GSh1 ***p* < 0.01, T98G Sh2 ***p* < 0.01).

D. ELISA detection of CXCL14 levels following knockdown (U87Sh1 ****p* < 0.001, U87Sh2 ****p* < 0.001; U251Sh1 ****p* < 0.001, U251Sh2 ****p* < 0.001; T98GSh1 ****p* < 0.001, T98G Sh2 ****p* < 0.001).

E. Wound healing assay in U87 and T98G cells following T-5224 treatment. Scale bar= 200μm.

F, G. Wound healing assay in U87 and T98G cells following FOS overexpression and knockdown. Scale bar= 200μm.

**
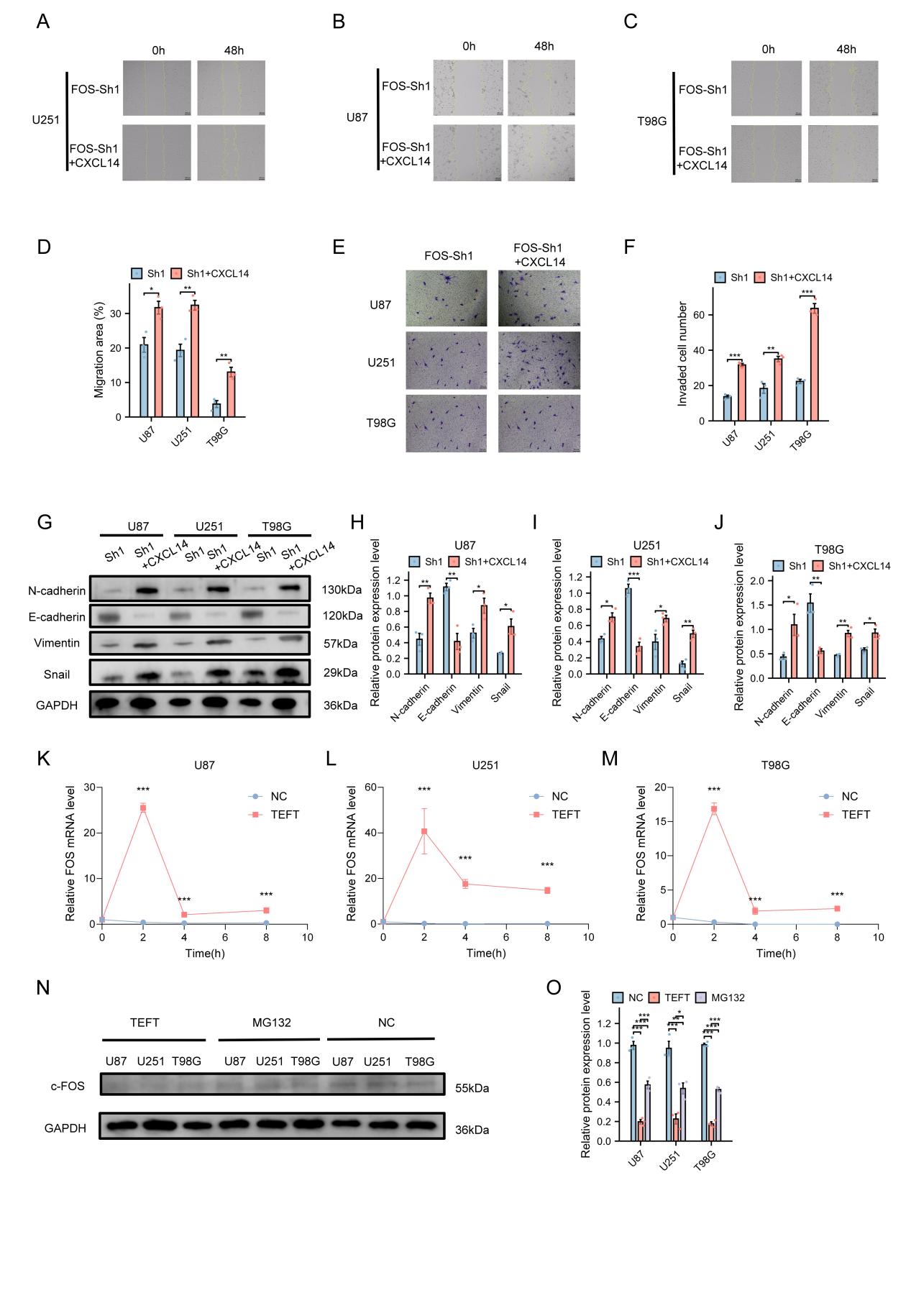
**

**Figure S6 c-FOS acts as an upstream regulator of CXCL14**

A-D. Wound healing assay results of U251, U87 and T98G cells with CXCL14 supplementation in FOS-Sh1 background and statistical charts across three cell lines, Student's *t*-test, n = 3, **p* < 0.05, ***p* < 0.01, ****p* < 0.001. Scale bar= 200μm.

E-F. Invasion assay and statistical chart of three cell lines with CXCL14 supplementation in FOS-Sh1 background, Student's *t*-test, n = 3, **p* < 0.05, ***p* < 0.01, ****p* < 0.001. Scale bar= 100μm.

G-J. WB Analysis and statistical chart of three cell lines with CXCL14 supplementation in FOS-Sh1 background, Student's *t*-test, n = 3, **p* < 0.05, ***p* < 0.01, ****p* < 0.001.

K-M. qRT-PCR results of U87, U251, and T98G cell lines after inhibiting mRNA with Actinomycin D, two-way ANOVA with Tukey’s post hoc test for two groups, n = 3, **p* < 0.05, ***p* < 0.01, ****p* < 0.001.

N-O. Western blot results in U87, U251, and T98G cell lines following treatment with the proteasome inhibitor MG-132. one-way ANOVA with Tukey’s post hoc test for three groups, n = 3, **p* < 0.05, ***p* < 0.01, ****p* < 0.001.

**Table S1**

The primer sequences used for qRT-PCR and ChIP assays are listed below:

| qPCR primers sequence (5' -> 3') |  |
| --- | --- |
| GAPDH Forward | GGAAGCTTGTCATCAATGGAAATC |
| GAPDH Reverse | TGATGACCCTTTTGGCTCCC |
| CXCL14 Forward | GAAATGAAGCCAAAGTACCCG |
| CXCL14 Reverse | CTTCTCGTTCCAGGCGTTGT |
| FOS Forward | GGGGCAAGGTGGAACAGTTA |
| FOS Reverse | AGGTTGGCAATCTCGGTCTG |
|  |  |
| Chip-qPCR primers sequence (5' -> 3') |  |
| CXCL14 Forward | GTGCGAAGGCGGCGTGTTGT |
| CXCL14 Reverse | TTAAATCCGCTCCTGCCCTC |

**Table S2**

The antibodies used in this study are listed as follows:

| Antibody | Application | Host | Supplier | dilution |
| --- | --- | --- | --- | --- |
| c-FOS | WB | Rabbit | Cat:# AF5354, Affinity Biosciences | 1:500 |
| CXCL14 | WB and IHC | Rabbit | Cat:# DF12377, Affinity Biosciences | 1:500 |
| E-cadherin | WB and IHC | Rabbit | Cat:# AF0131, Affinity Biosciences | 1:500 |
| N-cadherin | WB and IHC | Rabbit | Cat No. 22018-1-AP, proteintech | 1:2000 |
| Vimentin | WB and IHC | Mouse | Cat No. 60330-1-Ig ,proteintech | 1:20000 |
| Snail | WB | Rabbit | Cat:3879T, Cell Signaling technology | 1:1000 |
| GAPDH | WB | Mouse | Cat No. 60004-1-Ig,proteintech | 1:50000 |

**Table S3**

The detailed p-values are presented below:

| Figure | Experiment | Detailed *p*-values |
| --- | --- | --- |
| Figure 1J-M | WB | U87: N-cadherin *p* < 0.01, E-cadherin *p* < 0.001, Vimentin *p* < 0.001 Snail *p* < 0.05; U251: N-cadherin *p* < 0.01, E-cadherin *p* < 0.05, Vimentin *p* < 0.05, Snail *p* < 0.05; T98G: N-cadherin *p* < 0.05, E-cadherin *p* < 0.01, Vimentin *p* < 0.05, Snail *p* < 0.05 |
| Figure 1A-M | Wound healing assay  Transwell assays  WB | Wound healing assay: U87: 24h *p* < 0.05, 48h *p* < 0.05; U251: 24h *p* < 0.05, 48h *p* < 0.01; T98G: 24h *p* < 0.001 48h *p* < 0.001; Transwell assays: U87: *p* < 0.001; U251: *p* < 0.001; T98G: *p* < 0.001;WB: U87: N-cadherin *p* < 0.05, E-cadherin *p* < 0.05, Vimentin *p* < 0.01, Snail *p* < 0.001; U251: N-cadherin *p* < 0.01, E-cadherin *p* < 0.01, Vimentin *p* < 0.001, Snail *p* < 0.05; T98G: N-cadherin *p* < 0.01, E-cadherin *p* < 0.01, Vimentin *p* < 0.001, Snail *p* < 0.05 |
| Figure 3F-I | WB | U87: N-cadherin *p* < 0.001, E-cadherin *p* < 0.01, Vimentin *p* < 0.01, Snail *p* < 0.01; U251: N-cadherin *p* < 0.05, E-cadherin *p* < 0.05, Vimentin *p* < 0.001, Snail *p* < 0.01; T98G: N-cadherin *p* < 0.05, E-cadherin *p* < 0.01, Vimentin *p* < 0.01, Snail *p* < 0.05 |
| Figure S4A-D | qRT-PCR  ELISA | qRT-PCR: OE: U87: *p* < 0.01; U251: *p* < 0.05; T98G: *p* < 0.01; Sh: U87Sh1: *p* < 0.001, U87Sh2: *p* < 0.001; U251Sh1: *p* < 0.001, U251Sh2: *p* < 0.001; T98GSh1: *p* < 0.001, T98GSh2: *p* < 0.001; ELISA: OE: U87: *p* < 0.01; U251: *p* < 0.001; T98G: *p* < 0.01; Sh: U87Sh1: *p* < 0.01, U87Sh2: *p* < 0.01; U251Sh1: *p* < 0.01, U251Sh2: *p* < 0.01; T98GSh1: *p* < 0.01, T98GSh2: *p* < 0.01 |
| Figure 3J-M, Figure S4F, G | Wound healing assay | OE: U87: *p* < 0.01; U251: *p* < 0.01; T98G: *p* < 0.001; Sh: U87Sh1: *p* < 0.01, U87Sh2: *p* < 0.01; U251Sh1: p < 0.01, U251Sh2: p < 0.01; T98GSh1: p < 0.001, T98GSh2: p < 0.001 |
| Figure 3N-Q | Transwell assays | OE: U87: *p* < 0.001; U251: *p* < 0.001; T98G: *p* < 0.01; Sh: U87Sh1: *p* < 0.01, U87Sh2: *p* < 0.01; U251Sh1: *p* < 0.001, U251Sh2: *p* < 0.001; T98GSh1: *p* < 0.001, T98GSh2: *p* < 0.001 |
| Figure 3R-Y | WB | OE: U87: N-cadherin *p* < 0.05, E-cadherin *p* < 0.01, Vimentin *p* < 0.05, Snail *p* < 0.05; U251: N-cadherin *p* < 0.01, E-cadherin *p* < 0.01, Vimentin *p* < 0.05, Snail *p* < 0.01; T98G: N-cadherin *p* < 0.05, E-cadherin *p* < 0.05, Vimentin *p* < 0.001, Snail *p* < 0.01;Sh1: U87: N-cadherin *p* < 0.01, E-cadherin *p* < 0.01, Vimentin *p* < 0.001, Snail *p* < 0.05; U251: N-cadherin *p* < 0.01, E-cadherin *p* < 0.05, Vimentin *p* < 0.05, Snail *p* < 0.05; T98G: N-cadherin *p* < 0.001, E-cadherin *p* < 0.001, Vimentin *p* < 0.01, Snail *p* < 0.01; Sh2: U87: N-cadherin *p* < 0.01, E-cadherin *p* < 0.01, Vimentin *p* < 0.001, Snail *p* < 0.01; U251: N-cadherin *p* < 0.05, E-cadherin *p* < 0.05, Vimentin *p* < 0.05, Snail *p* < 0.01; T98G: N-cadherin *p* < 0.001, E-cadherin *p* < 0.001, Vimentin *p* < 0.01, Snail *p* < 0.001 |
| Figure 5E-H | WB | U87: N-cadherin *p* < 0.001, E-cadherin *p* < 0.05, Vimentin *p* < 0.01, Snail *p* < 0.05; U251: N-cadherin *p* < 0.01, E-cadherin *p* < 0.01, Vimentin *p* < 0.05, Snail *p* < 0.05; T98G: N-cadherin *p*< 0.01, E-cadherin *p* < 0.001, Vimentin *p* < 0.05, Snail *p* < 0.01 |
| Figure 5Q-R, Figure S5A-D | qRT-PCR  WB  ELISA | qRT-PCR: OE: U87: *p* < 0.001; U251: *p* < 0.001; T98G: *p* < 0.001; Sh: U87Sh1: *p* < 0.001, U87Sh2: *p* < 0.001; U251Sh1: *p* < 0.001, U251Sh2: *p* < 0.01; T98GSh1: *p* < 0.01, T98GSh2: *p* < 0.01; WB: OE：U87: *p* < 0.05; U251: *p* < 0.01; T98G: *p* < 0.05；Sh: U87Sh1: *p* < 0.001, U87Sh2: *p* < 0.001; U251Sh1: *p* < 0.01, U251Sh2: *p* < 0.01; T98GSh1: *p* < 0.01, T98GSh2: *p* < 0.01; ELISA: OE：U87: *p* < 0.001; U251: *p* < 0.001; T98G: *p* < 0.001；Sh: U87Sh1: *p* < 0.001, U87Sh2: *p* < 0.001; U251Sh1: *p* < 0.001, U251Sh2: *p* < 0.001; T98GSh1: *p* < 0.001, T98GSh2: *p* < 0.001 |
| Figure 5I-L Figure S5F, G | Wound healing assay | OE: U87: *p* < 0.01; U251: *p* < 0.05; T98G: *p* < 0.01; Sh: U87Sh1: *p* < 0.001, U87Sh2: *p* < 0.001; U251Sh1: *p* < 0.05, U251Sh2: *p* < 0.01; T98GSh1: *p* < 0.01, T98GSh2: *p* < 0.001 |
| Figure 5M-P | Transwell assays | OE: U87: *p* < 0.001; U251: *p* < 0.001; T98G: *p* < 0.01; Sh: U87Sh1: *p* < 0.01, U87Sh2: *p* < 0.001; U251Sh1: *p* < 0.001, U251Sh2: *p* < 0.01; T98GSh1: *p* < 0.05, T98GSh2: *p* < 0.01 |
| Figure 5Q-X | WB | OE: U87: N-cadherin *p* < 0.05, E-cadherin *p* < 0.05, Vimentin *p* < 0.05, Snail *p* < 0.05; U251: N-cadherin *p* < 0.05, E-cadherin *p* < 0.05, Vimentin *p* < 0.05, Snail *p* < 0.05; T98G: N-cadherin *p* < 0.01, E-cadherin *p* < 0.001, Vimentin *p* < 0.05, Snail *p* < 0.01;Sh1: U87: N-cadherin *p* < 0.05, E-cadherin *p* < 0.05, Vimentin *p* < 0.001, Snail *p* < 0.05; U251: N-cadherin *p* < 0.05, E-cadherin *p* < 0.01, Vimentin *p* < 0.01, Snail *p* < 0.01; T98G: N-cadherin *p* < 0.01, E-cadherin *p* < 0.05, Vimentin *p* < 0.05, Snail *p* < 0.05; Sh2: U87: N-cadherin *p* < 0.01, E-cadherin *p* < 0.05, Vimentin *p* < 0.001, Snail *p* < 0.05; U251: N-cadherin *p* < 0.05, E-cadherin *p* < 0.01, Vimentin *p* < 0.01, Snail *p* < 0.01; T98G: N-cadherin *p* < 0.01, E-cadherin *p* < 0.01, Vimentin *p* < 0.01, Snail *p* < 0.01 |
| Figure S6A-H | Wound healing assay  Transwell assays  WB | Wound healing assay:U87: *p* < 0.05; U251: *p* < 0.01; T98G: *p* < 0.01; Transwell assays: U87: *p* < 0.001; U251: *p* < 0.01; T98G: *p* < 0.001; WB: U87: N-cadherin *p* < 0.01, E-cadherin *p* < 0.01, Vimentin *p* < 0.05, Snail *p* < 0.05; U251: N-cadherin *p* < 0.05, E-cadherin *p* < 0.001, Vimentin *p* < 0.05, Snail *p* < 0.01; T98G: N-cadherin *p* < 0.05, E-cadherin *p* < 0.01, Vimentin *p* < 0.01, Snail *p* < 0.05 |
| Figure 6I, J | IHC | OE: CXCL14 *p* < 0.01, N-cadherin *p* < 0.05, E-cadherin *p* < 0.001, Vimentin *p* < 0.05; Sh1: CXCL14 *p* < 0.05, N-cadherin *p* < 0.05, E-cadherin *p* < 0.05, Vimentin *p* < 0.01 |
