# Supplementary material for: Tumor Electric Field Therapy Inhibits Epithelial‐Mesenchymal Transition, Invasion, and Migration of Glioblastoma by Targeting the c‐FOS/CXCL14 Axis
Source: CNS Neurosci Ther. 2026 May 19;32(5):e70926. doi: 10.1002/cns.70926 (PMC13185563; doi:10.1002/cns.70926)
Supplement: Supplementary file 2 — Supporting Information Methods. A comprehensive description of all experimental procedures, materials, and analytical methods is provided in the Supplementary Methods document. [file CNS-32-e70926-s002.docx]

***Supplementary Methods***

**GBM cell line culture**

**The U251, U87, and T98G cell lines employed in this study were acquired from the Institute of Basic Medicine, China Medical College. For cell cultivation, Dulbecco's Modified Eagle's Medium with high glucose (DMEM, Gibco) supplemented with 10% fetal bovine serum (FBS, Gibco) and 1% penicillin-streptomycin (Servicebio, G4003)** **was utilized as the culture medium. All cell culture processes were conducted in a cell incubator maintained at a constant temperature of 37°C and a CO₂ concentration of 5%** ^[7, 10]^**.**

**GBM cell lines exposed on TEFT**

**GBM cell lines exposed on TEFT process refers to previous articles published by our research group** ^[7, 10]^**. GBM cells were plated onto 20-mm glass slides (Nest 801008) at a density of 2 × 10⁵ cells/mL, with 150 μL of cell suspension added to each slide. Following an overnight incubation period to allow for cell adhesion, the cells were subjected to electric field treatment using a TEFT device (CL-301A)** ^[30]^ **at a frequency of 200 kHz and a field strength of 2.2 V/m for 72 hours. Control groups were cultured under otherwise identical conditions but without exposure to TEFT.**

**Collection of TEFT related animal and clinical specimens**

**This study was approved by the Institutional Review Board of PLA General Hospital (approval number: 2018-089-01). Written informed consent was obtained from all enrolled patients. For immunohistochemical (IHC) staining, three pairs of paraffin-embedded GBM tissue samples were utilized, which were collected from patients both prior to and following TEFT intervention. Furthermore, GBM samples derived from rats that had undergone TEFT were retrieved from our previous research for subsequent analyses** ^[31]^**.**

**Transcriptional sequencing of U251 cells**

**To identify differentially expressed genes (DEGs), we treated U251 cells with TGF-β1 and compared them against a nc group. Total RNA was isolated from these cells using Trizol reagent (Takara) in strict accordance with the manufacturer’s recommended protocol. Subsequently, polyadenylated (poly(A)) RNAs and non-coding RNAs (ncRNAs) were purified from the extracted total RNA via oligo(dT) beads. The enriched RNAs were used for the construction of RNA sequencing (RNA-seq) libraries, and sequencing was performed on an Illumina platform at Majorbio Corporation. For preprocessing of raw sequencing reads, adapter sequences were trimmed and reads with low complexity or poor quality were filtered out to obtain clean reads. These clean reads were then aligned to the human reference genome assembly GRCh38.p13 using the HISAT2 aligner. The datasets used and/or analyzed during the current study are available from the corresponding author on reasonable request.**

**Analysis transcriptional data of U251 cells**

**DEGs were identified using the DESeq2 (version 1.36.0) and edgeR R package (version 3.38.2). The screening criteria for DEGs were set as an absolute log₂ fold change (FC) > 1.5 and an adjusted *P*-value < 0.05. Among these identified DEGs, those with a log₂ FC > 1.5 were categorized as up-regulated DEGs, which are indicative of increased gene expression levels. In contrast, DEGs with a log₂ FC < -1.5 were classified as down-regulated DEGs, corresponding to decreased gene expression. Volcano plot constructed by ggplot2 R package (version 3.4.4) was used to present DEGs.**

**Acquisition multi-omics of GBM**

**Gene expression profiles and corresponding clinical data of glioma samples were retrieved from the GlioVis portal (**<http://gliovis.bioinfo.cnio.es/>**)** ^[32]^**, which included multiple datasets: TCGA-GBM (HG-U133A and Agilent-4502A), Gravendeel, Bao, LeeY, Rembrandt and Ivy GAP datasets. IHC staining results of glioma tissue sections were obtained from the Human Protein Atlas (HPA) database (**<https://www.proteinatlas.org/>**)** ^[33]^**. Additionally,** **reverse-phase protein array (RPPA) data of TCGA-GBM specimens were also extracted from GlioVis. Single-cell RNA sequencing data of GBM were accessed *via* the Tumor Immune Single-cell Hub 2 (TISCH2) database (**<http://tisch.comp-genomics.org/>**)** ^[34]^**. The GSE131928 dataset was analyzed to investigate the expression pattern of the CXCL14 gene across different GBM cell subpopulations. For the aggregated single-cell expression data, dimensionality reduction was performed using the uniform manifold approximation and projection (UMAP) algorithm. Furthermore, Gene Set Enrichment Analysis (GSEA) was employed to quantitatively assess key phenotypes. The top 100 mesenchymal-like (MES-like) malignant genes were obtained for further analysis.**

**Screening the hub gene related to EMT and TEFT anti-GBM effect**

**The candidate gene set was defined as the intersection of three gene subsets: the top 100 MES-like malignant genes, down-regulated DEGs in the TEFT group of the T98G cell line identified in our previous study**^[10]^**, and up-regulated DEGs in the TGF-β1 group of the U251 cell line. Furthermore, survival analysis and gene expression analysis stratified by GBM subtypes (MES, PN and CL) were constructed to further characterize these candidates. The hub genes were screened based on the fulfillment of three stringent criteria. First, they must be members of the candidate gene set. Second, it must be prognostic-related genes in GBM. Third, they must exhibit the highest expression level in the MES subtype among all GBM subtypes.**

**Proteogenomics exploration of CXCL14**

**The LinkedOmicsKB database was utilized to obtain proteogenomic data of GBM and pan-cancer (https://kb.linkedomics.org/)** ^[35]^**. Association analysis of the protein abundance of CXCL14 with various phenotypes was conducted to uncover the potential functions of CXCL14. For exploring the relationship between CXCL14 and EMT, GSEA was performed on the protein level of CXCL14 in both GBM and pan-cancer contexts. Additionally, association analysis of CXCL14 multi-omics data (mRNA, protein and methylation level) in GBM was carried out to reveal the key regulatory mechanisms of CXCL14 in this disease.**

**Prediction transcription factors of CXCL14**

**The TF-Target Finder (https://jingle.shinyapps.io/TF_Target_Finder/) which contains datasets such as Chip_Atlas, GTRD, CHEA, ENCODE, PWMEnrich_JASPAR, FIMO_JASPAR, and KnockTF, was used to screen for potential transcription factors (TFs) of CXCL14. The Log_2_ FC for KnockTF was set at 0.5. TFs presented in at least four datasets were identified as potential TFs using upset plot. Results related to TCGA-GBM from correlation analysis module were applied to determine whether TFs are correlated with CXCL14 at transcriptional level. Finally, the HPA database was employed to confirm whether the potential TFs are expressed in glioma on protein level.**

**Wound healing and transwell assay**

**Cells were seeded into 6-well plates. A sterile 200 μL pipette tip was used to create a linear scratch wound, and the cells were washed twice with PBS to remove debris before being cultured in serum-free DMEM. Wound healing was monitored at 0, 24 and 48h using an inverted microscope, and the migration distance was measured and analyzed using ImageJ software (version 1.54p). For the transwell migration assay, 1 × 10⁴ cells were resuspended in serum-free medium and seeded in the upper chamber of transwell inserts with an 8 μm pore size (Corning, 354480). The lower chamber was filled with 600 μL of DMEM containing 10% FBS as a chemoattractant. After 24 h of incubation at 37 °C in 5% CO₂, non-migrated cells on the upper surface of the membrane were gently removed with a cotton swab. Migrated cells on the lower surface were fixed with 4% paraformaldehyde for 15 min, stained with 0.1% crystal violet for 20 min, and counted under an inverted microscope (Olympus BX51, Japan) in three randomly selected fields. For the transwell invasion assay, the procedure was identical, except the transwell inserts were precoated with Matrigel (Corning, 356234) to mimic the ECM. All experiments were performed in triplicate.**

**Quantitative real-time PCR**

**Total RNA was extracted from cell samples using the RNA extraction solution (Servicebio, G3013). cDNA synthesis was performed with SweScript All-in-One RT SuperMix for Quantitative real-time PCR (qRT-PCR) (Servicebio, G3337). The qRT-PCR was carried out on the Applied Real-Time PCR System (BioRad,** CFX Connect**) under the following conditions: 95°C for 30 seconds, then 40 cycles of 95°C for 15 seconds and 60°C for 1 minute. Each reaction was run in triplicate, using GAPDH as the reference gene. Relative target gene expression was calculated via the 2-****ΔΔCt method. Primer sequences used can be found in Table S1.**

**Western Blot (WB)**

**Proteins were extracted from samples using RIPA lysis buffer (Servicebio, G2002) supplemented with protease inhibitor Cocktail (Servicebio, G2006) and PMSF (Servicebio, G2008). The extracted proteins were separated by SDS-PAGE on 4%-12% polyacrylamide gels and transferred onto polyvinylidene difluoride (PVDF) membranes. Membranes were blocked with 5% skim milk prepared in Tris-buffered saline containing 0.1% Tween-20 (TBST) for 1 h at room temperature, followed by incubation with primary antibodies specific to the target proteins overnight at 4°C. After thorough washing with TBST, membranes were incubated for 1 h at room temperature with horseradish peroxidase- conjugated secondary antibodies (1:5000 dilution). Protein bands were visualized using enhanced chemiluminescence reagents, and band intensities were quantified using ImageJ software. Antibodies used can be found in Table S2.**

**Chromatin Immunoprecipitation (CHIP)**

**For the ChIP assay, cells were crosslinked with 1% formaldehyde in the culture medium, and the reaction was quenched with glycine. Cells were harvested using a scraper and lysed in ChIP lysis buffer. Chromatin was digested with micrococcal nuclease to generate DNA fragments of approximately 150–900 bp. After reserving 2% of the sample as input, the remaining chromatin solution was incubated with the specific primary antibody or IgG negative control overnight at 4 °C. Protein G Agarose Beads were added to the chromatin solution and incubated for 2 h at 4 °C. The chromatin was then eluted from the antibody/Protein G Agarose Beads and de-crosslinked. Finally, DNA was purified and quantified by qPCR. Primers used for ChIP assays are listed in Table S1.**

**Immunofluorescence staining (IF)**

**U87, U251 and T98G cells were harvested during log phase growth. After trypsinization to detach adhered cells, the cell suspension was centrifuged, and the cell pellet was resuspended in fresh medium. The cells were counted and seeded onto laser confocal culture dishes (Nest, 801001) at the appropriate density. The cells were incubated for 3 hours to allow adhesion. Then, cultured overnight in 1 mL of medium added after the adhesion period. The culture medium was aspirated, and the cells were washed with 1 × PBS. The cells were then fixed at room temperature for 15 min using 4% paraformaldehyde solution. The cells were permeabilized with 0.3% Triton X-100 solution for 20 min followed by blocking with 5% BSA for 1 h. Primary antibody incubation was performed overnight at 4°C using a 1:200 dilution. After washing, the cells were incubated for 1.5 h at room temperature with fluorophore-conjugated secondary antibody diluted at 1:500, while protected from light. Nuclei staining was done using DAPI for 10 min in the dark. Finally, the prepared cell samples were imaged under a laser-scanning confocal microscope (Olympus, FV1000).**

**Construction of stable knockdown and overexpression GBM lines**

**Stable knockdown and overexpression models of CXCL14 and FOS genes were established in GBM cell lines. The shRNA sequences targeting CXCL14 and FOS genes along with overexpression constructs were professionally designed and synthesized by Tsingke Biotechnology (Beijing). HEK293T cells were transfected using Lipofectamine 2000 to pack viral particles. The viral supernatant was collected and used to infect U87, U251, and T98G cells. Twenty-four hours post-infection, the culture medium was replaced with selection medium containing 4 μg/mL puromycin according to the manufacturer's instructions, followed by continuous culture for 2-3 weeks until single-cell colonies emerged. After expansion, total RNA and proteins were extracted to verify the knockdown and overexpression efficiency of target genes through qRT-PCR and WB. Monoclonal cell lines with significantly altered gene expressions were selected, successfully establishing stable knockdown or overexpressing GBM cell lines.**

**Enzyme-Linked Immunosorbent Assay (ELISA)**

**The concentration of target protein in cell culture supernatants or tissue lysates was quantified using a commercial ELISA kit (Servicebio, GEH0014) according to the manufacturer's instructions. Briefly, 100 μL of standards and samples were added to the antibody-precoated wells and incubated for 2 hours at 37°C. After washing three times with 300 μL of provided wash buffer, 100 μL of biotinylated detection antibody was added to each well and incubated for 1 hour at 37°C. Following another three washes, 100 μL of horseradish peroxidase (HRP)-conjugated streptavidin was added and incubated for 30 minutes at 37°C protected from light. After a final five washes, 100 μL of tetramethylbenzidine (TMB) substrate was added and incubated for 15 minutes at room temperature. The reaction was stopped by adding 50 μL of stop solution, and the absorbance was immediately measured at 450 nm using a microplate reader (BioTeK, Epoch). All samples were assayed in triplicate, and the protein concentration was determined by interpolating from the standard curve.**

**mRNA Stability Assay**
**To evaluate the stability of FOS mRNA, cells were seeded in 6-well plates and allowed to reach 70–80% confluence before receiving indicated treatments. Briefly, the transcriptional inhibitor Actinomycin D** **(MCE, [HY-17559](https://www.medchemexpress.cn/Actinomycin-D.html)) was added to the culture medium at a final concentration of 10 μg/mL to arrest de novo mRNA synthesis**^[36]^**. Total RNA was then harvested at specified time intervals (0, 2, 4 and 8 hours) following Actinomycin D treatment using TRIzol reagent. The mRNA levels of target genes were quantified by RT-qPCR as described above.**

**Proteasome Inhibition and Protein Stability Assay**
**To determine whether the degradation of c-FOS is dependent on the ubiquitin-proteasome pathway, cells were seeded in 6-well plates and grown to 70–80% confluence. Cells were subjected to indicated treatments in the presence or absence of the specific proteasome inhibitor MG132 (20 μM; MCE, [HY-13259](https://www.medchemexpress.cn/MG-132.html)) for 8 hours**^[37]^**.**

**Immunohistochemistry**

**The tissues were fixed in 4% paraformaldehyde solution, embedded in paraffin, and sectioned into 4-μm-thick slices. The tissue sections were mounted on slides and processed following the previously described protocol** ^[7]^**.The tissue sections were incubated overnight at 4°C with a primary antibody against CXCL14 (Affnity, DF12377, 1:1000 dilution) and c-FOS (Affnity, AF5354, 1:1000 dilution) in 1% goat serum (Balb, WE0320) PBS solution. After washing, the sections were incubated for 1 h at room temperature with the appropriate secondary antibody. The tissue slices were then stained using the ABC Horseradish Peroxidase kit (Vector Laboratories) and 3,3'-diaminobenzidine (DAB) as the chromogen for visualization. Hematoxylin counterstaining was performed to visualize nuclei. Two pathologists, blinded to clinical information on the samples, independently evaluated and scored the resulting immunohistochemical staining patterns.**

**Intracranial in *situ* tumorigenic mouse model**

**Five-week-old female athymic BALB/c nude mice were purchased from GemPharmatech Co., Ltd. (Guangdong, China) and acclimatized for one week prior to the experiments. All animal care and experimental procedures were conducted in accordance with the animal welfare protocol approved by Servicebio (Protocol No. 2025227). The mice were randomly divided into three groups with six mice per group. U251 wild-type cells, FOS-knockdown cells, and FOS-overexpressing cells, all of which were modified to express firefly luciferase via lentiviral transduction, were suspended in phosphate-buffered saline at a density of 1×10⁶ cells in 2 μL and stereotactically injected into the mouse brain using a microsyringe. The injection coordinates were set at 1.8 mm right and 0.49 mm anterior to the bregma, with a needle depth of 3.5 mm. Tumor growth and development were monitored and quantified using the IVIS Spectrum *in vivo* imaging system. To analyze tumor progression and invasion, brain tissues were harvested 30 days post-injection, fixed in 4% paraformaldehyde, and embedded in paraffin for hematoxylin and eosin (HE) staining and IHC analysis.**

**Statistical Analysis**

**The Shapiro–Wilk test was utilized to assess the normality of the data distribution. For continuous variables following a normal distribution, the Student's t-test was utilized for comparisons between two groups, while one-way ANOVA with Tukey's post hoc test was used for comparisons across multiple groups. For non-normally distributed continuous data, the non-parametric Wilcoxon rank-sum test was used for two group comparisons. The Kruskal–Wallis test followed by post-hoc Dunn's multiple comparisons was utilized for comparisons across multiple groups. Comparison of Kaplan–Meier survival curves was accomplished using the Log-rank test. Patients were stratified into high (high-expression group: 50%–100%) and low (low-expression group: 0%–50%)-expression groups based on median mRNA levels. The survival R package (version 3.3.1) was utilized for survival statistics and survminer (version 3.3.6) for visualization. R software (version 4.2.1) was utilized to perform statistical analyses. p-values less than 0.05 from two-sided tests were considered statistically significant.**
